# Supplementary material for: Apical and Basal Matrix Remodeling Control Epithelial Morphogenesis
Source: Dev Cell. 2018 Jul 2;46(1):23–39.e5. doi: 10.1016/j.devcel.2018.06.006 (PMC6035286; doi:10.1016/j.devcel.2018.06.006)
Supplement: Document S1. Figures S1–S7 [file mmc1.pdf]

Developmental Cell, Volume *46*

Supplemental Information

Apical and Basal Matrix Remodeling

Control Epithelial Morphogenesis

Maria-del-Carmen Diaz-de-la-Loza, Robert P. Ray, Poulami S. Ganguly, Silvanus Alt, John R. Davis, Andreas Hoppe, Nic Tapon, Guillaume Salbreux, and Barry J. Thompson

Supplemental material:

Supplemental Figures S1-S7

Figure S1. Broad, MMP1 and Ultrabithorax localisation from late larvae to early pupal stages.

Figure S2. Analysis of cell anisotropy, cell rearrangements and cell area during convergent extension and expansion.

Figure S3. Quantification of Myosin-II localisation and a continuum model of convergent extension in the pupal wing.

Figure S4. The Fat cadherin system and the polarity protein Bazooka are not essential for anisotropic extension of the pupal wing.

Figure S5. Perlecan localisation in 7 hours APF legs.

Figure S6. Myosin-II localisation in the leg and the haltere.

Figure S7. Cellular attachment to the basal ECM and concomitant detachment from the apical ECM leads to the folding of the epithelia.

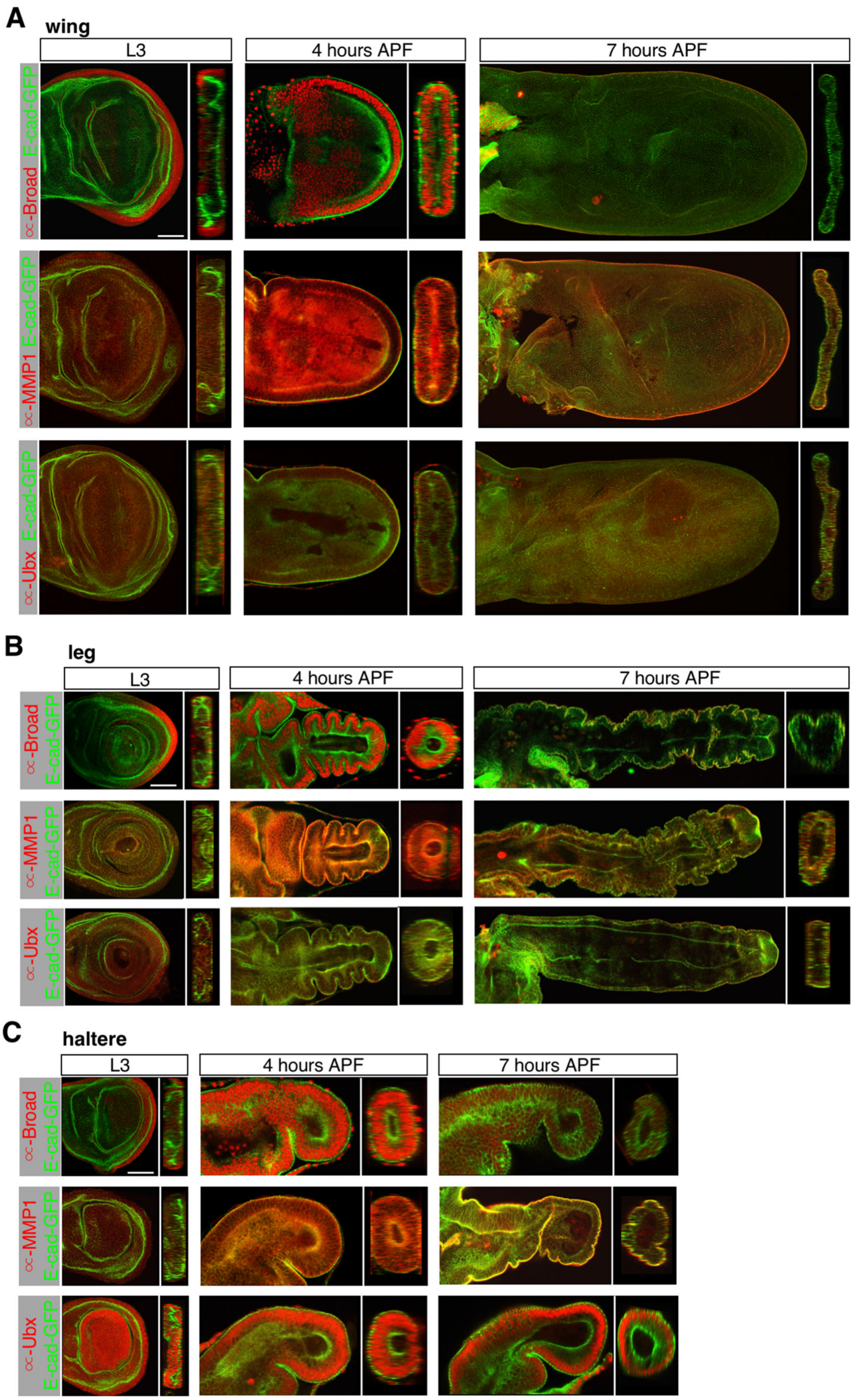

Figure S1. Broad, MMP1 and Ultrabithorax localisation from late larvae to early pupal stages. Related to Figure 2. Cross sections of developing wings (A), legs (B) and halteres (C), at third instar larvae stages (L3), 4 and 7 hours APF expressing *E-cadherin-GFP* (E-cad-GFP). Expression of *broad*, *MMP1* metalloprotease, and *Ultrabithorax* (*Ubx*) was detected by immunostaining. *broad* and *MMP1* are strongly expressed in all imaginal disc epithelia at 4 hours APF, but are almost no detectable at 7 hours APF. *Ubx* is exclusively expressed in the haltere epithelia. Although basal extracellular matrix (ECM) is not degraded in the haltere, MMP1 can be detected at 4 hours APF, suggesting that MMP1 activity should be inhibited in that tissue, most likely by the tissue inhibitor of metalloproteases (Timp). E-cad-GFP is shown in green and Broad, MMP1 and Ubx are shown in red. Scale bar, 50 µm.

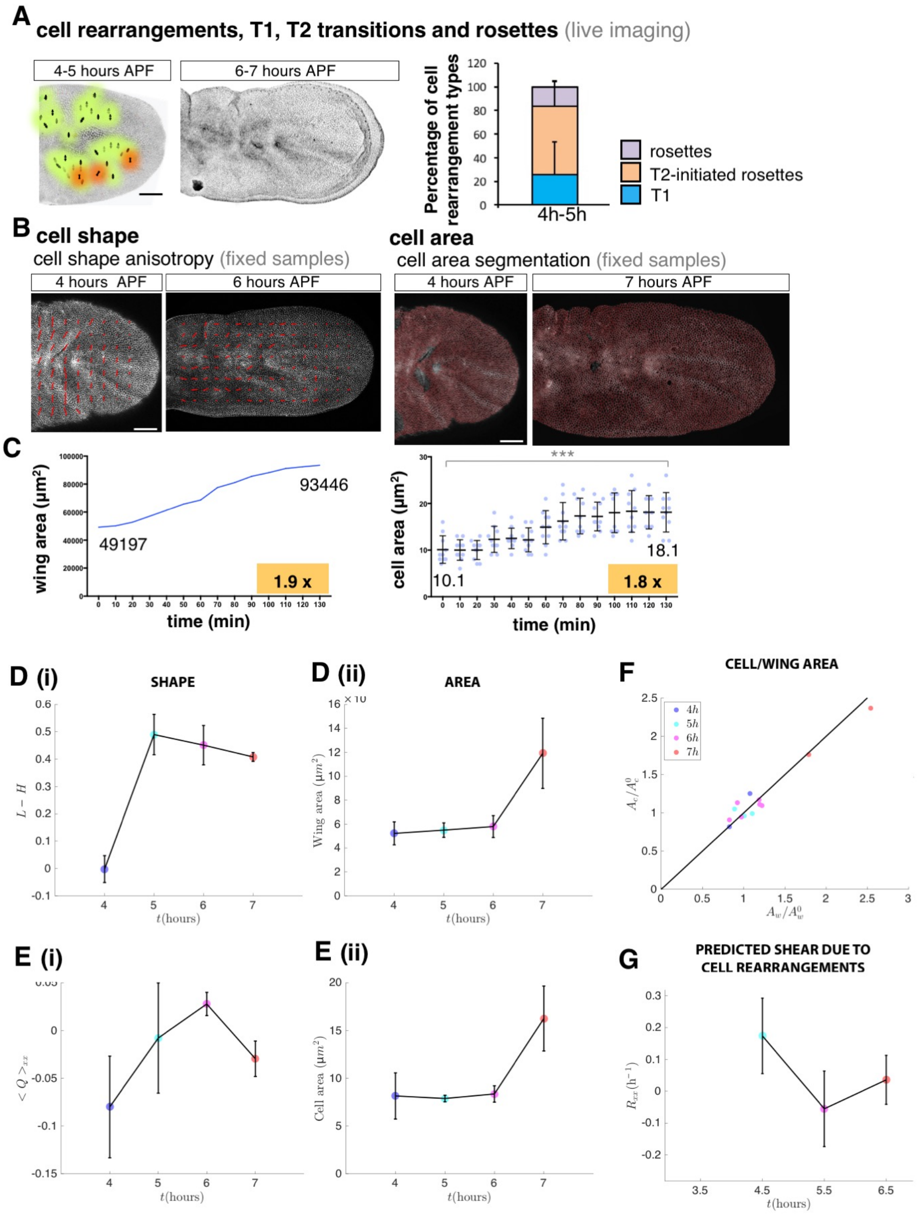

Figure S2. Analysis of cell anisotropy, cell rearrangements and cell area during convergent extension and expansion. Related to Figure 3.

(A-B) Cell rearrangements and cell shape and cell size changes were detected by live imaging experiments in *E-cad-GFP* expressing wings. (A) Cell intercalations were only present during convergent extension, mostly consisting in the shrinkage of junctions in the same direction of wing contraction through T1, T2 and rosettes that were often initiated after a T2 transition event (right, green shadowed arrows; red arrows indicate intercalation in the opposite direction). Average and standard deviation are represented for each type of cell rearrangement, n=4 wings, >1500 cells per wing. (B) Cell size and shape were analysed through segmentation on E-cad-GFP fixed samples from 4 to 7 hours APF. Red lines indicate the intensity and the direction of cell anisotropy (left) and the cell apical perimeter (right). Scale bar, 50 µm (left).

(C) Quantification of cell area increase during wing expansion in live imaging experiments, from 6 to 8 hours APF (Movie S1). Average and standard deviation of apical cell area (n=10) are represented, and statically significant differences are indicated (***p<0.001).

(D) Quantification of wing shape (i) and area (ii). Anisotropy of the wing, measured in natural strain variables $L - H$, increases from 4 hours to 5 hours APF and remains roughly constant after 5 hours. Wing area increases slightly from 4 to 6 hours APF and increases greatly from 6 to 7 hours APF.

(E) Quantification of shape (i) and area (ii) of wing epithelia segmented cells. (A i) Average cell elongation $< Q_{xx} >$ increases sharply from 4 to 5 hours APF, indicating the cells are elongated along the anterior-posterior axis at the wing disc at 4 hours become almost isotropic at 5 hours APF. Cell areas are approximately constant from 4 to 5 hours APF, increase slightly from 5 to 6 hours APF, and sharply from 6 to 7 hours APF.

(F) The increase in cell area is sufficient to explain the increase in wing area. Normalised cell areas, (A_c / A_c^0), increase linearly with normalised wing areas, (A_w / A_w). A_c, cell area; A_c^0, average cell area at 5 hours APF; A_w, wing areas; A_w^0, average wing area at 5 hours APF. Each point (colour coded according to development time) corresponds to a single wing disc sample.

(G) The contribution of topological transitions to the overall convergent extension of the wing given by $R_{xx} = \frac{1}{2}\frac{d(L-H)}{dt} - \frac{d\langle Q_{xx}\rangle}{dt}$. R_{xx} is most pronounced from 4 to 5 hours APF and accounts for approximately half of the increase in anisotropy of the wing, the other half being due to change in cell elongation (see figure S3 and Supplemental Material and Methods). The data was obtained by averaging 2, 3, 6 and 2 fixed wings disc samples at 4, 5, 6 and 7 hours APF, respectively. The error bars correspond to standard deviation among wing discs at the same time point in development.

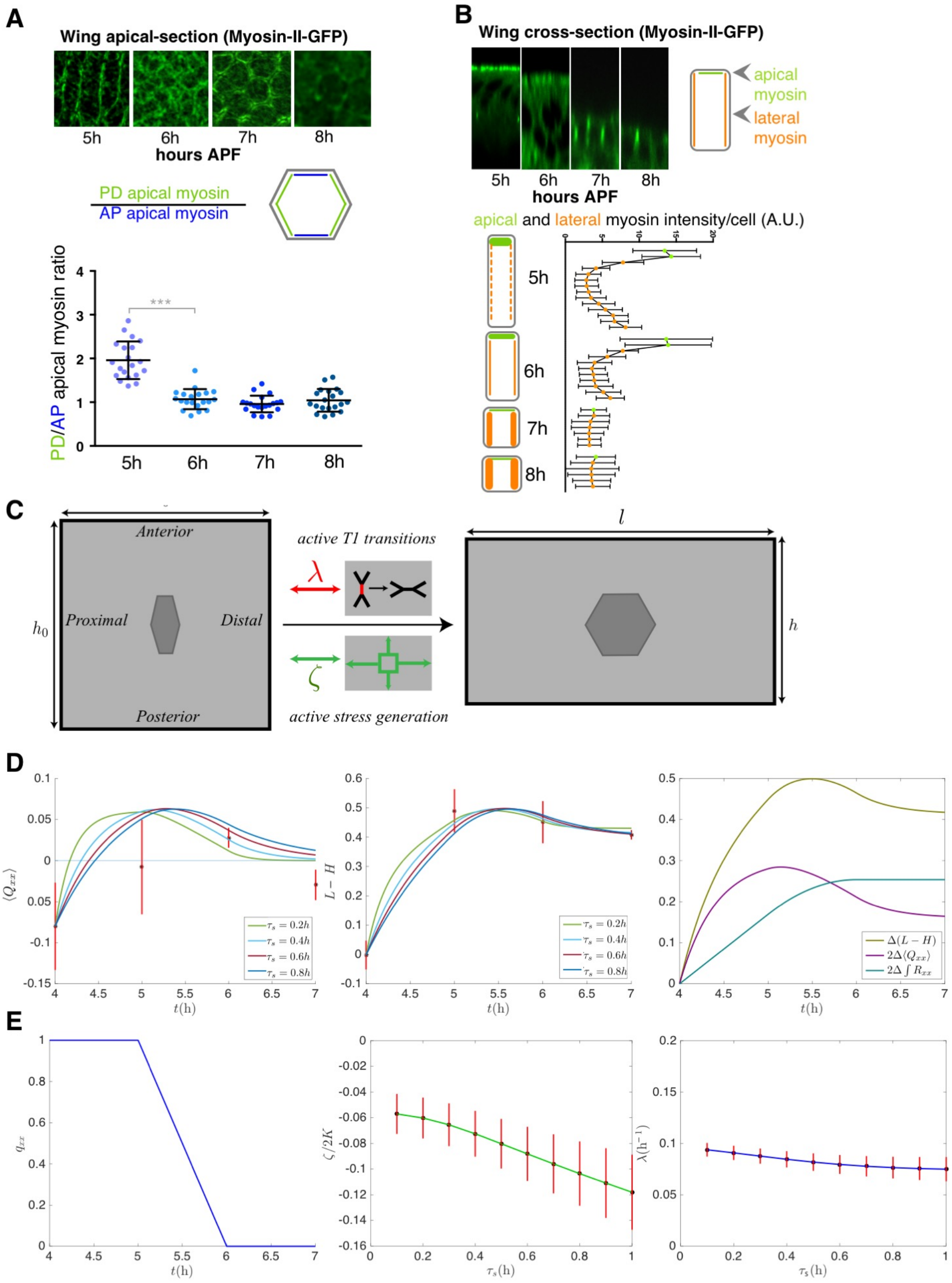

Figure S3. Quantification of Myosin-II localisation and a continuum model of convergent extension in the pupal wing (see Supplemental Material and Methods). Related to Figure 3 and 4.

(A) Measurements of Myosin-II-GFP fluorescence intensity were taken in Fiji at various points around the apical cell-cell junction of wing epithelia at the times shown. The ratio of myosin levels along the proximal-distal (PD) axis and anterior-posterior (AP) axis of the wing was calculated and plotted for multiple samples and time points. Note the 2-fold initial polarisation of Myosin-II-GFP along the proximal-distal axis at 5 hours (h) APF, which later dissipates. n=20 for each developmental stage. Average, standard deviation, and individual data points are represented. Statistically significant differences are indicated (***p<0.001).

(B) Measurements of Myosin-II-GFP fluorescence intensity were taken in Fiji at various points along the apical-basal axis of wing epithelia at the times shown. Data were plotted as intensity levels coloured by apical versus basolateral localisation as shown. At the end of convergent extension (5 to 6 hours APF), the apical surface shows the maximum values for Myosin-II-GFP fluorescence, whereas at the end of expansion and flattening, fluorescence levels are equally distributed along the apico-basal axis. n=20 for each developmental stage. Average and standard deviation are represented.

(C) The wing is approximated by a rectangular piece of tissue that deforms over time. Active stress generation due to polarised myosin in the tissue and oriented active topological transitions are responsible for the anisotropic growth of the wing.

(D) Time evolution of (*left*) the average cell anisotropy ($\langle Q_{xx}\rangle$) and (*centre*) the wing anisotropy ($L-H$) obtained from a model of tissue deformation. Theory curves are fitted to experimental data (black points with red standard deviation bars) by taking different values for τ_s, the viscoelastic relaxation time of the tissue. *(right)* The contributions of cell shape change ($2\Delta\langle Q_{xx}\rangle$) and topological transitions ($2\Delta\int R_{xx}$) to the overall change in wing anisotropy $\Delta(L-H)$ for $\tau_s = 0.4$ h.

(E) (*left*) Time evolution of cell polarity q_{xx} taken for the fit, (*centre*) fitted parameter values of $\frac{\zeta}{2K}$ and (*right*) λ for a range of τ_s (red bars, standard error).

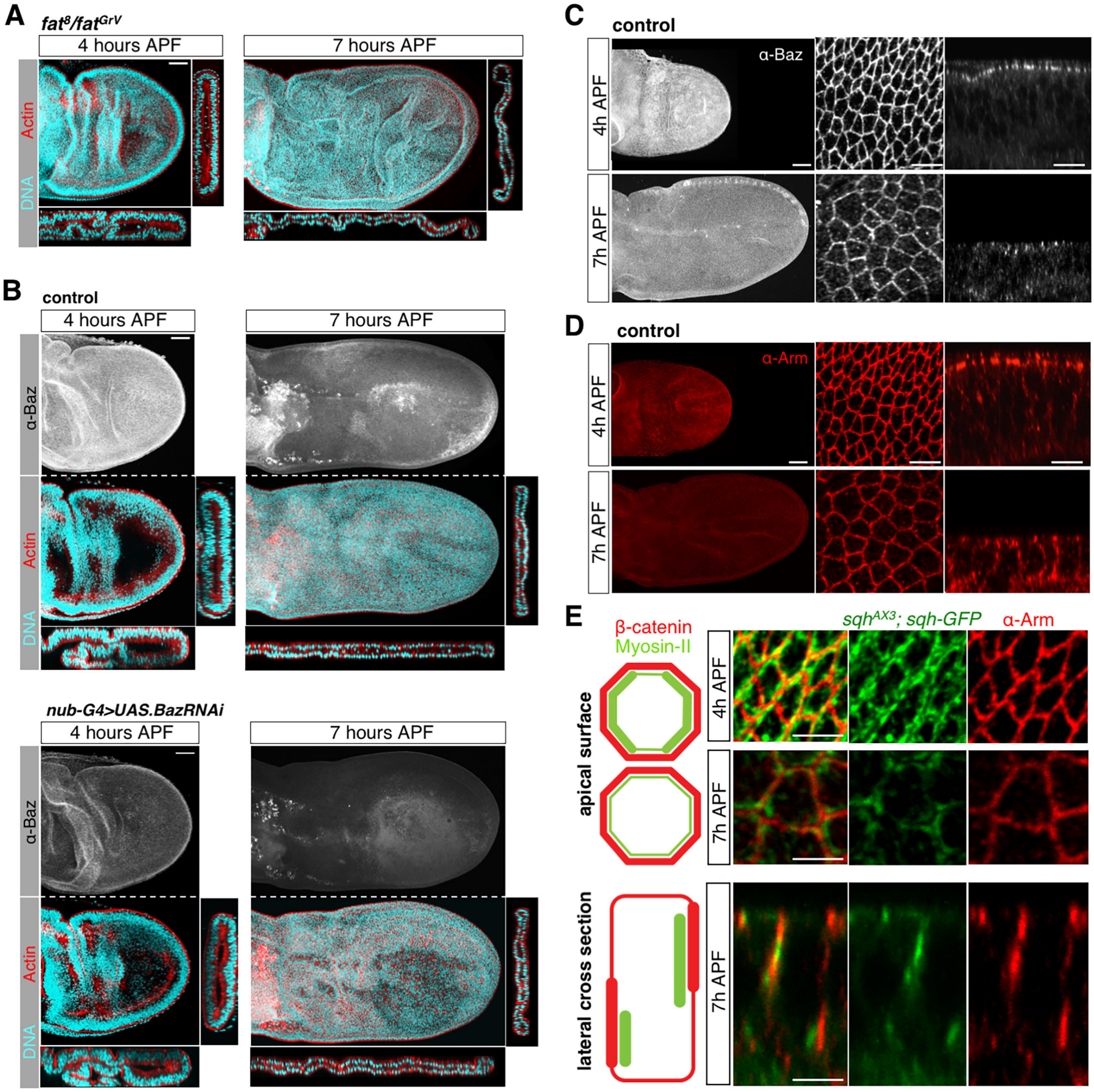

Figure S4. The Fat cadherin system and the polarity protein Bazooka are not essential for anisotropic extension of the pupal wing. Related to Figure 4.

(A) Inactivation of the Ds/Fat/Dachs planar polarity system in the *fat*^{8}/*fat*^{GrV} mutant (top). The *fat* mutant wings are already rounded prior to expansion and elongation, indicating that the key role of this planar polarity pathway is during larval stages of development, rather than during pupal stages. Scale bar 50 μm.

(B) Depletion of Bazooka/Par6 (Baz) in *nub-G4>UAS.bazRNAi* decreases Bazooka protein levels in the wing but does not affect anisotropic elongation of the epithelia. Control (top) and *nub-G4>UAS.bazRNAi* (bottom) wings showing Baz immunostaining in grey, actin in red and nuclei (DNA) in blue. Scale bars are 50 μm.

(C) In contrast to the elongating embryo, in which Bazooka/Par6 is planar polarised (Zallen and Wieschaus, 2004), Baz is not planar polarised during wing elongation, where it instead localises to adherens junctions. Scale bars are 50 μm (left) and 10 μm (middle and right).

(D) Armadillo/beta-catenin (Arm) co-localises with Bazooka in an apical ring and also localises laterally during expansion at 7 hours APF. Scale bars are 50 μm (left) and 10 μm (middle and right). Scale bars are 50 μm.

(E) Myosin-II localises adjacent to the adherens junctions marked by Arm both at the apical surface at 4 hours APF, and at the lateral membrane from 7 hours APF. Scale bar, 10 μm.

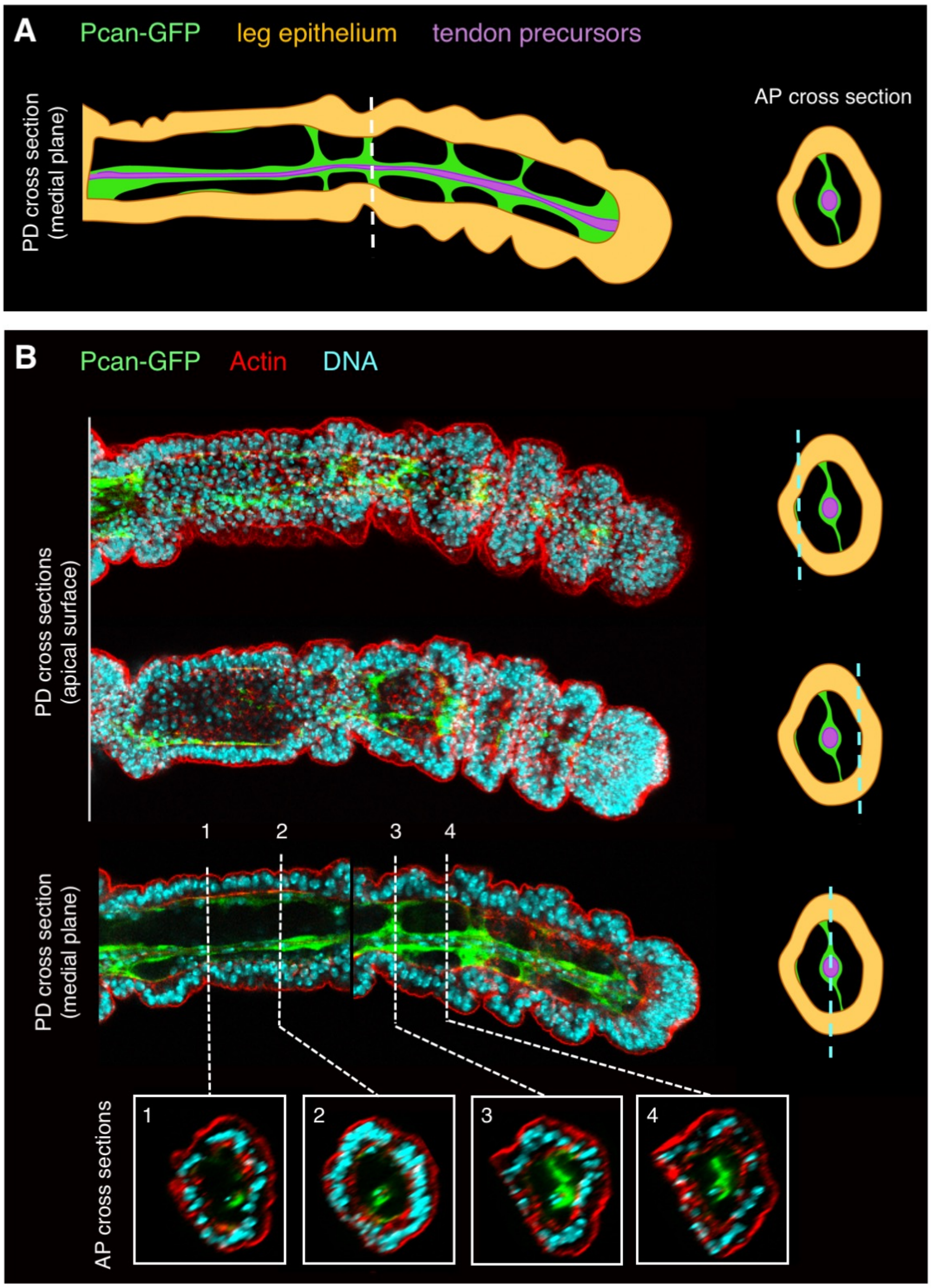

Figure S5. Perlecan localisation in 7 hours APF legs. Related to Figure 6.
(A) Diagram of a 7 hours APF leg. A dense layer of Perlecan (Pcan-GFP, green) covers the long tendon precursor that runs within the appendage (purple), but is less abundant at the basal side of the leg epithelium (yellow).
(B) Proximal-distal (PD) and anterior-posterior (AP) cross sections of 7 hours APF developing leg expressing *Pcan-GFP*. Traces of Pcan-GFP are present in the apical surface of the leg epithelium, but mostly accumulates surrounding then tendon precursors.

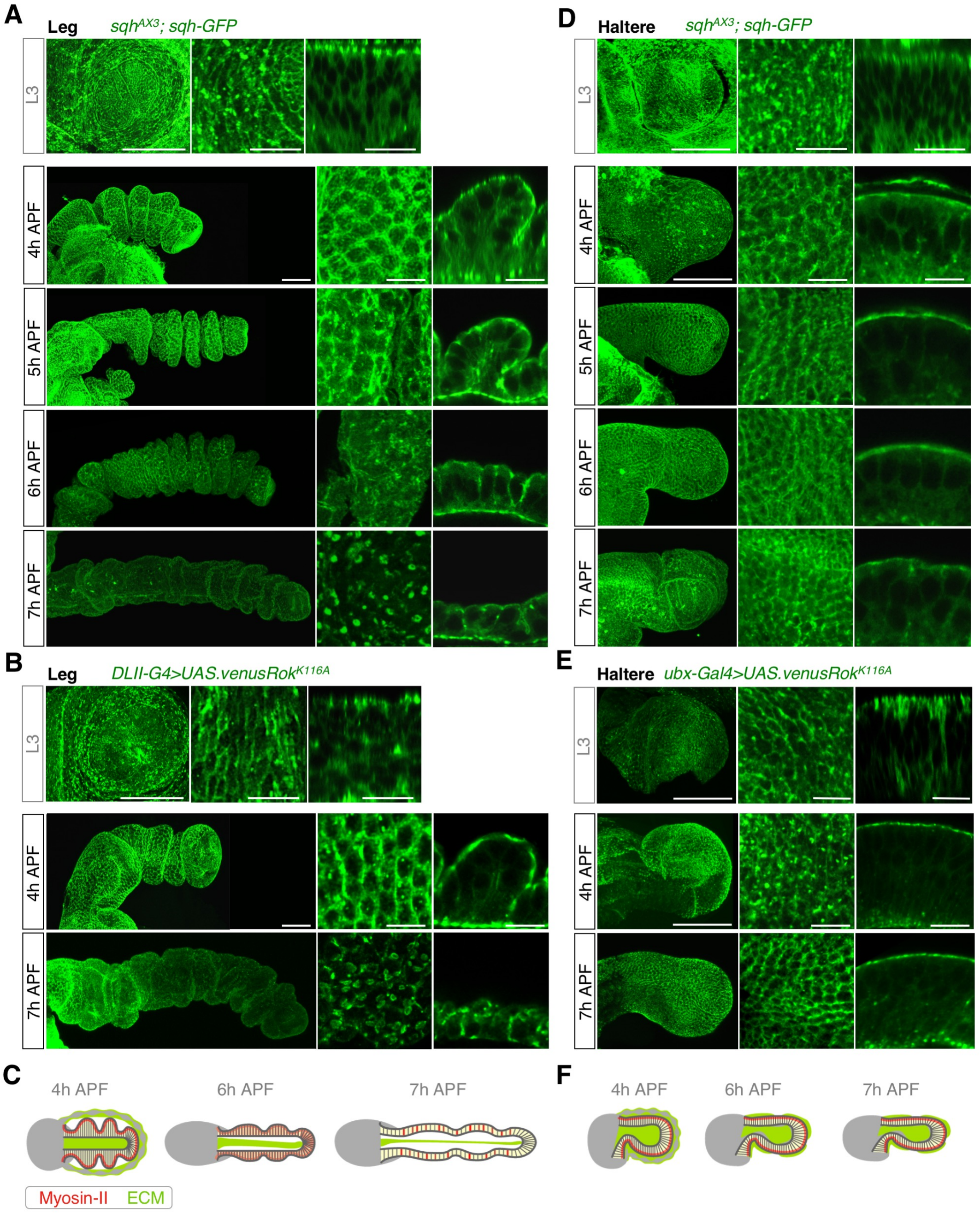

Figure S6. Myosin-II localisation in the leg and the haltere. Related to Figures 4, 5 and 6.

(A) Myosin-II-GFP localisation in the developing *Drosophila* leg. Maximum projections (left), high-magnification apical view XY-sections (middle) and lateral view Z-sections (right) of *sqhAX3; {sqh-GFP}* imaginal discs are shown. As in the wing, apical Myosin-II re-localises laterally in the leg epithelia from 5 to 7 hours APF.

(B) Similarly to Myosin-II, GFP-Rok is planar polarised in the leg at late larval stages, but localises laterally during the expansion phase (from 6 to 8 hours AF). Scale bars: 50µm (left) and 10µm (mid and right).

(C) Schematic diagram of leg disc morphogenesis from at 4 to 7 hours APF in sagittal cross sections. Leg epithelia is shown in yellow and peripodial membrane in grey. Apical and basal ECM is shown in green, and Myosin-II in red. Similarly to the wing, after peripodial membrane release and apical and basal ECM degradation, Myosin-II starts to re-localise from apical to the lateral plasma membrane, that, together with the unfolding of the leg, contributes to the flattening of the epithelia and the elongation of the leg discs.

(D) Myosin-II-GFP localisation in the developing *Drosophila* haltere. Maximum projections (left), high-magnification apical view XY-sections (middle) and lateral view Z-sections (right) of *sqhAX3; {sqh-GFP}* imaginal discs are shown. In contrast with wing and leg imaginal discs, Myosin-II remains apical in the haltere from L3 to 7 hours APF.

(E) Similarly to Myosin-II, GFP-Rok remains apical in the haltere from L3 to 7 hours APF.

(F) Schematic diagram of haltere disc morphogenesis from at 4 hours to 7 hours APF in sagittal cross sections. Haltere epithelia is shown in yellow and peripodial membrane in grey. Apical and basal ECM is shown in green, and Myosin II in red. In contrast with the wing and the leg, Myosin-II is localised apically from 4 to 7 hours APF, the epithelia barely flatten and the entire structure does not elongate.

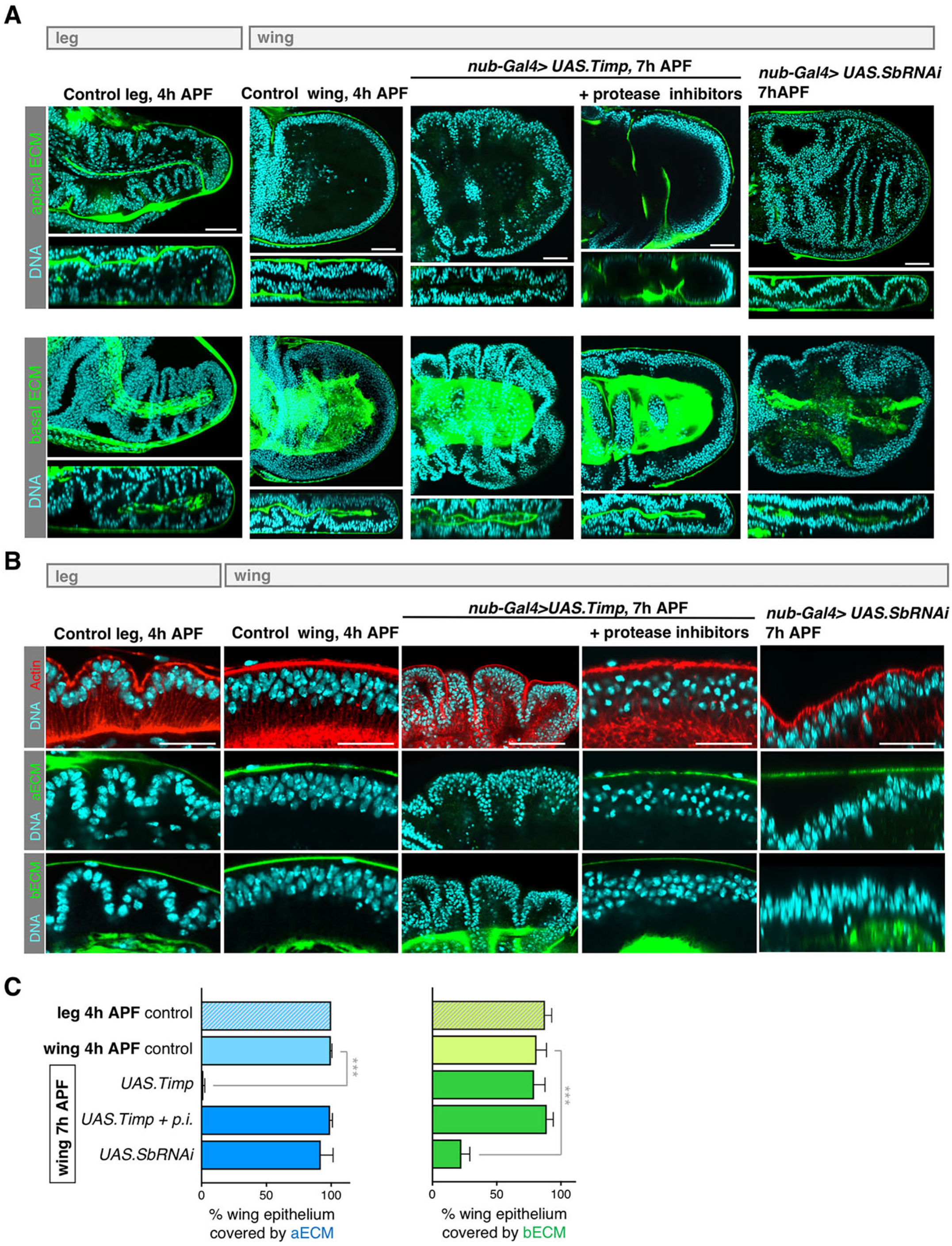

Figure S7. Cellular attachment to the basal ECM and concomitant detachment from the apical ECM leads to the folding of the epithelia. Related to Figure 1, 2 and 5.

(A) Cross sections of 4 hours APF control leg and wing, 7 hours APF *Timp* overexpressing wings (*dp-YFP nub-Gal4>UAS.Timp*) with or without protease inhibitor treatment, and *Stubble* overexpressing wing (*nub-Gal4>UAS.SbRNAi*). *Timp* overexpression does not affect basal Dumpy-YFP release but completely inhibits basal Collagen IV-GFP degradation, which impairs wing expansion and folds the epithelia. Protease inhibitors impair Dumpy release in *Timp* overexpressing wings and rescues the epithelial folding. Scale bar, 50 µm.

(B) High magnification of folded epithelia in 4 hours APF control leg and 7 hours APF wings overexpressing *Timp* or *Stubble*, and straight columnar epithelia in 4 hours APF control wing and 7 hours APF *Timp* overexpressing wings treated with protease inhibitors. When cells are attached to the apical but not the basal ECM it results in the folding of the epithelia. Scale bar, 25 µm.

(C) Quantification of the percentage of epithelia covered with apical Dumpy-YFP and basal Collagen IV-GFP in the different tissues and experimental conditions shown in (A,B). Average and standard deviation are represented, n≥4 for each developmental stage. Statistically significant differences between the control and the experimental conditions are indicated (***p<0.001).
