# Supplementary material for: Apical and Basal Matrix Remodeling Control Epithelial Morphogenesis
Source: Dev Cell. 2018 Jul 2;46(1):23–39.e5. doi: 10.1016/j.devcel.2018.06.006 (PMC6035286; doi:10.1016/j.devcel.2018.06.006)
Supplement: Document S2. Article plus Supplemental Information [file mmc9.pdf]

Developmental Cell

Article

Apical and Basal Matrix Remodeling Control Epithelial Morphogenesis

Graphical Abstract

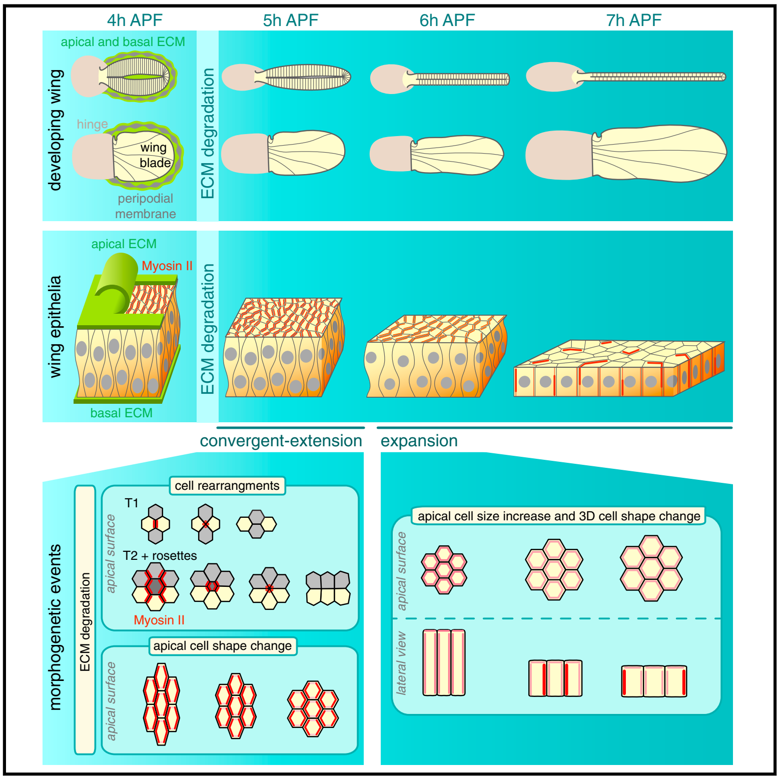

Authors

Maria-del-Carmen Diaz-de-la-Loza, Robert P. Ray, Poulami S. Ganguly, ..., Nic Tapon, Guillaume Salbreux, Barry J. Thompson

Correspondence

barry.thompson@crick.ac.uk

In Brief

Diaz-de-la-Loza et al. show that morphogenetic elongation of *Drosophila* limbs occurs via both convergent extension and columnar-to-cuboidal cell shape change. These processes are spatially organized by Myosin-II and temporally organized by remodeling of the extracellular matrix, including both apical (ZP-domain-containing) and basal (Collagen IV/Laminin/Perlecan-containing) matrices.

Highlights

- Apical and basal extracellular matrices are degraded to elongate *Drosophila* limbs
- Apical matrix is degraded by the Stubble protease and basal matrix by MMPs
- Limbs elongate via convergent extension and cell flattening, driven by Myosin-II
- In the haltere, Ultrabithorax prevents matrix remodeling and tissue elongation

Diaz-de-la-Loza et al., 2018, Developmental Cell *46*, 23–39
July 2, 2018 © 2018 Elsevier Inc.
https://doi.org/10.1016/j.devcel.2018.06.006

CellPress

Apical and Basal Matrix Remodeling Control Epithelial Morphogenesis

Maria-del-Carmen Diaz-de-la-Loza,[1] Robert P. Ray,[5] Poulami S. Ganguly,[2] Silvanus Alt,[2,6] John R. Davis,[3] Andreas Hoppe,[4] Nic Tapon,[3] Guillaume Salbreux,[2] and Barry J. Thompson[1,7,*]
[1]Epithelial Biology Laboratory, The Francis Crick Institute, 1 Midland Road, London NW1 1AT, UK
[2]Theoretical Physics of Biology Laboratory, The Francis Crick Institute, 1 Midland Road, London NW1 1AT, UK
[3]Apoptosis and Proliferation Control Laboratory, The Francis Crick Institute, 1 Midland Road, London NW1 1AT, UK
[4]Kingston University, Penrhyn Road, Kingston upon Thames, London KT1 2EE, UK
[5]HHMI Janelia Research Campus, 19700 Helix Drive, Ashburn, VA 20147, USA
[6]Max-Delbrück Center for Molecular Medicine, Robert-Rössle-Straße 10, Berlin-Buch 13125, Germany
[7]Lead Contact
*Correspondence: barry.thompson@crick.ac.uk
https://doi.org/10.1016/j.devcel.2018.06.006

SUMMARY

Epithelial tissues can elongate in two dimensions by polarized cell intercalation, oriented cell division, or cell shape change, owing to local or global actomyosin contractile forces acting in the plane of the tissue. In addition, epithelia can undergo morphogenetic change in three dimensions. We show that elongation of the wings and legs of *Drosophila* involves a columnar-to-cuboidal cell shape change that reduces cell height and expands cell width. Remodeling of the apical extracellular matrix by the Stubble protease and basal matrix by MMP1/2 proteases induces wing and leg elongation. Matrix remodeling does not occur in the haltere, a limb that fails to elongate. Limb elongation is made anisotropic by planar polarized Myosin-II, which drives convergent extension along the proximal-distal axis. Subsequently, Myosin-II relocalizes to lateral membranes to accelerate columnar-to-cuboidal transition and isotropic tissue expansion. Thus, matrix remodeling induces dynamic changes in actomyosin contractility to drive epithelial morphogenesis in three dimensions.

INTRODUCTION

The generation of form in living organisms is one of the most fascinating unsolved problems in biology (Dreher et al., 2016; Pasakarnis et al., 2016). Genetic analysis of epithelial tissue morphogenesis in model organisms has revealed that epithelia can elongate by either polarized cell intercalation (Pare et al., 2014; Blankenship et al., 2006; Bertet et al., 2004; Zallen and Wieschaus, 2004; Heisenberg et al., 2000; Irvine and Wieschaus, 1994; Keller, 1980) or oriented cell division (Campinho et al., 2013; Gibson et al., 2011; Mao et al., 2011; da Silva and Vincent, 2007; Baena-Lopez et al., 2005; Gong et al., 2004; Wei and Mikawa, 2000; Concha and Adams, 1998). These two general mechanisms for elongation of epithelial sheets are also observed during elongation of epithelial tubules in *Drosophila* and vertebrates (Saxena et al., 2014; Lienkamp et al., 2012; Saburi et al., 2008; Voiculescu et al., 2007). Both epithelial cell intercalation or oriented cell division can be driven either by local forces arising from planar polarized Myosins or by global forces acting across entire tissues (Collinet et al., 2015; Etournay et al., 2015; Lye et al., 2015; Ray et al., 2015; Legoff et al., 2013; Mao et al., 2013; Lye and Sanson, 2011; Vichas and Zallen, 2011; Lecuit and Le Goff, 2007).

A third general mechanism of epithelial morphogenesis is cell shape change. Recent research has been focused mainly on forces acting to shape the apical domain in two dimensions (Dreher et al., 2016; Pasakarnis et al., 2016; Paluch and Heisenberg, 2009). However, epithelial cells can also undergo three-dimensional shape changes to drive morphogenesis. One example is the columnar-to-cuboidal shape change that reduces apical-basal cell height and expands the apical surface to drive expansion and elongation of the *Drosophila* wing and leg (Fristrom and Fristrom, 1975; Poodry and Schneiderman, 1970). This mechanism was found to be intrinsic to the tissue itself, rather than driven by external forces, as it can occur *ex vivo* (Fristrom, 1988; Fristrom and Fristrom, 1975). Later work identified similar cell shape flattening events occurring during embryonic development of the fishes *Fundulus heteroclitus* and *Danio rerio*, the frog *Xenopus laevis*, and the sea anemone *Nematostella vectensis*, indicating that this morphogenetic mechanism is widespread in the animal kingdom (Fritz et al., 2013; Behrndt et al., 2012; Fristrom, 1988; Keller and Trinkaus, 1987; Keller, 1980). How columnar-to-cuboidal shape change might be developmentally controlled remains poorly understood.

One possible control mechanism has been observed in the *Drosophila* wing and leg, where an overlying layer of cells known as the peripodial (“around the foot”) layer is removed and discarded prior to the onset of columnar-to-cuboidal shape change and tissue elongation (Fristrom, 1988; Milner et al., 1984). The removal of the peripodial layer was found to be driven by Myosin-II contractility in the peripodial cells (Aldaz et al., 2013), yet whether removal of this layer is strictly causative for the subsequent wing expansion and elongation remains unclear. Here we show that remodeling of the extracellular matrix (ECM), rather

Check for updates

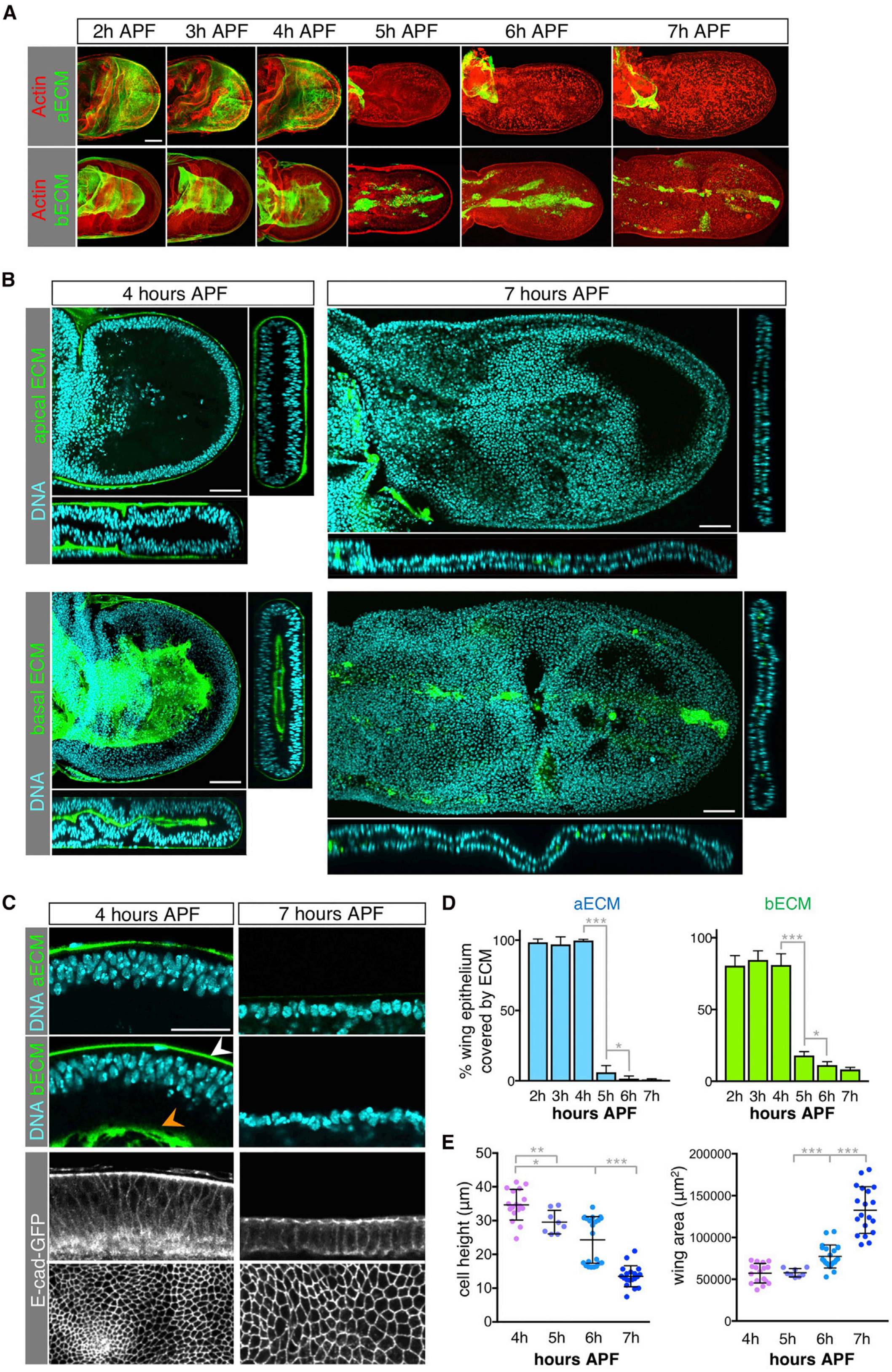

(legend on next page)

than removal of peripodial cells, is the causative event responsible for the initiation of *Drosophila* wing elongation, followed by columnar-to-cuboidal cell shape change to drive tissue expansion. First, ECM degradation triggers convergent extension to elongate the wing anisotropically and once that is achieved the tissue can perform the final event of flattening and expansion, growing isotropically by a decrease in cell height that increases cell width. Wing elongation involves planar polarization of Myosin-II, which induces convergent extension, followed by relocalization of Myosin-II laterally with respect to the apico-basal polarity of the cell, which then drives columnar-to-cuboidal transition and isotropic tissue expansion. Finally, we show that matrix remodeling is also necessary for leg elongation, but does not occur in the haltere, a homologous limb that fails to elongate despite removal of the peripodial layer. The decision of halteres not to undergo matrix remodeling and consequent expansion and extension is controlled by the homeobox gene *Ultrabithorax*.

RESULTS

Matrix Remodeling Controls Columnar-to-Cuboidal Cell Shape Change, which Drives Tissue Expansion and Elongation

We began by applying modern live-imaging methods (Aldaz et al., 2010) to reproduce the seminal work of early investigators who characterized the morphogenesis of the *Drosophila* wing and leg epithelia by transmission electron microscopy (Fristrom and Fristrom, 1975; Mandaron, 1970, 1971; Poodry and Schneiderman, 1970). Imaging of GFP-tagged E-cadherin (E-cad-GFP) confirms their key finding that morphogenetic expansion and elongation of the wing occurs by columnar-to-cuboidal cell shape change, a process that flattens the wing as it increases in both length and width (Figures 1A–1C). The key events take place between 4 and 7 hr after puparium formation (APF), prior to cuticle secretion, when the tall pseudo-stratified columnar epithelial cells become dramatically shorter along their apical-basal axis, such that initially densely packed nuclei become neatly aligned side by side and the apical area of each cell expands (Figures 1B and 1C).

To identify the mechanism controlling columnar-to-cuboidal cell shape change in the elongating *Drosophila* wing, we considered the possible role of the ECM. Previous work has implicated the basal Collagen IV-based ECM in the process of maintaining columnar cell shape in the wing (Pastor-Pareja and Xu, 2011). Since the wing epithelium also produces an apical ECM composed of the ZP-domain protein Dumpy, which has an important role in attachment of the epithelium to the exoskeleton at later stages of development (Ray et al., 2015), we examined the distribution of both types of matrix during the process of wing expansion and elongation. We have analyzed the localization of fluorescent-tagged versions of ECM components: apical Dumpy (Dumpy-YFP, Dp-YFP); and the main components of the basal matrix, Collagen IV (Collagen IV α2-subunit, encoded by *viking*, Vkg-GFP). We found that both apical and basal matrices remain in place while wing cells are still columnar at or before 4 hr APF, but that both matrices start to be degraded at 5 hr APF, immediately prior to wing expansion and elongation until 7 hr APF. The apical ECM is completely removed whereas some basal ECM remnants are still present at 7 hr APF (Figure 1). Notably, the cuticle is not secreted until after wing convergent extension and expansion has completed (after 7 hr APF); therefore, Dumpy does not mediate the interaction of the epithelium with the chitinous exoskeleton during these morphogenetic events. Instead, Dumpy indeed participates as a component of the apical ECM to constrain the entire tissue. Our findings show that columnar cell shape correlates with the presence of the matrix and that the absence of matrix correlates with the acquisition of cuboidal cell shape, such that initially densely packed nuclei become neatly aligned side by side and the apical area of each cell expands (Figures 1C–1E).

To test whether matrix remodeling is necessary for wing elongation, we cultured wings *ex vivo* in the presence of a protease inhibitor cocktail, and found that the tissue remains encapsulated in both apical and basal ECM and fails to elongate at 7 hr APF (Figures 2A–2C). We found that the key protease responsible for degrading the apical ECM is the Stubble protease, which is related to the human sperm acrosin protease that degrades the egg zona pellucida (apical ECM) (Appel et al., 1993; Beaton et al., 1988). Depletion of Stubble expression impairs degradation of the ZP-domain protein Dumpy, and therefore inhibits wing elongation and expansion (Figures 2A and 2C). In the case of the basal Collagen IV ECM, the MMP1 and MMP2 proteases are required for limb extension and expansion, as shown by inhibition of their activity with the tissue inhibitor of metalloproteinases (Timp) (Godenschwege et al., 2000) (Figures 2B and 2C). Thus, matrix remodeling is essential for columnar-to-cuboidal shape change and wing morphogenesis.

We next sought to analyze the temporal control of ECM degradation during wing morphogenesis. At the end of larval stages, a pulse of the steroid hormone ecdysone induces pupariation and

Figure 1. Remodeling of the Extracellular Matrix Precedes Wing Elongation

(A) Time-lapse imaging of the apical extracellular matrix (ECM) component Dumpy-YFP (Dp-YFP, aECM) and basal ECM component Collagen IV-GFP (Collagen IV α2-subunit, Vkg-GFP, bECM), which distribute homogeneously in the developing wing from 2 to 4 hr (h) after puparium formation (APF), but are degraded from 5 hr APF, concomitant with peripodial membrane release. The entire wing disc is shown for Dp-YFP, and only the basal surface for Vkg-GFP. Scale bar, 50 μm.

(B) Cross-sections of developing wings at 4 and 7 hr APF. At 4 hr APF, Dp-GFP and Vkg-GFP cover the apical and the basal surface of the wing epithelia, respectively and Vkg-GFP also surrounds the basal side of the peripodial membrane. At 7 hr APF the matrix has been removed and only some traces of Vkg-GFP are still detected. Nuclei (DNA) are shown in blue. Scale bar, 50 μm.

(C) High-magnification view of epithelial cells showing that, at 4 hr APF, columnar epithelial cells distributed in a pseudo-stratified epithelium are in contact with apical Dp-YFP and basal Vkg-GFP (orange arrowhead), whereas by 7 hr APF the matrix is absent and cells adopt a more cuboidal morphology. The layer of Vkg-GFP that covers the basal surface of the peripodial membrane at 4 hr APF is indicated by a white arrowhead. Scale bar, 25 μm.

(D) Quantification of the percentage of epithelium covered with apical Dp-YFP and basal Vkg-GFP in developing wings from 2 to 7 hr APF. Average and SD are presented; $n > 4$ for each developmental stage. Statistically significant differences are indicated (*$p < 0.05$, ***$p < 0.001$).

(E) Quantification of wing blade area and epithelial cell height from 4 to 7 hr APF. Average, SD, and individual data points are presented; $n > 8$ for each developmental stage. Statistically significant differences are indicated (*$p < 0.05$, **$p < 0.005$, ***$p < 0.001$).

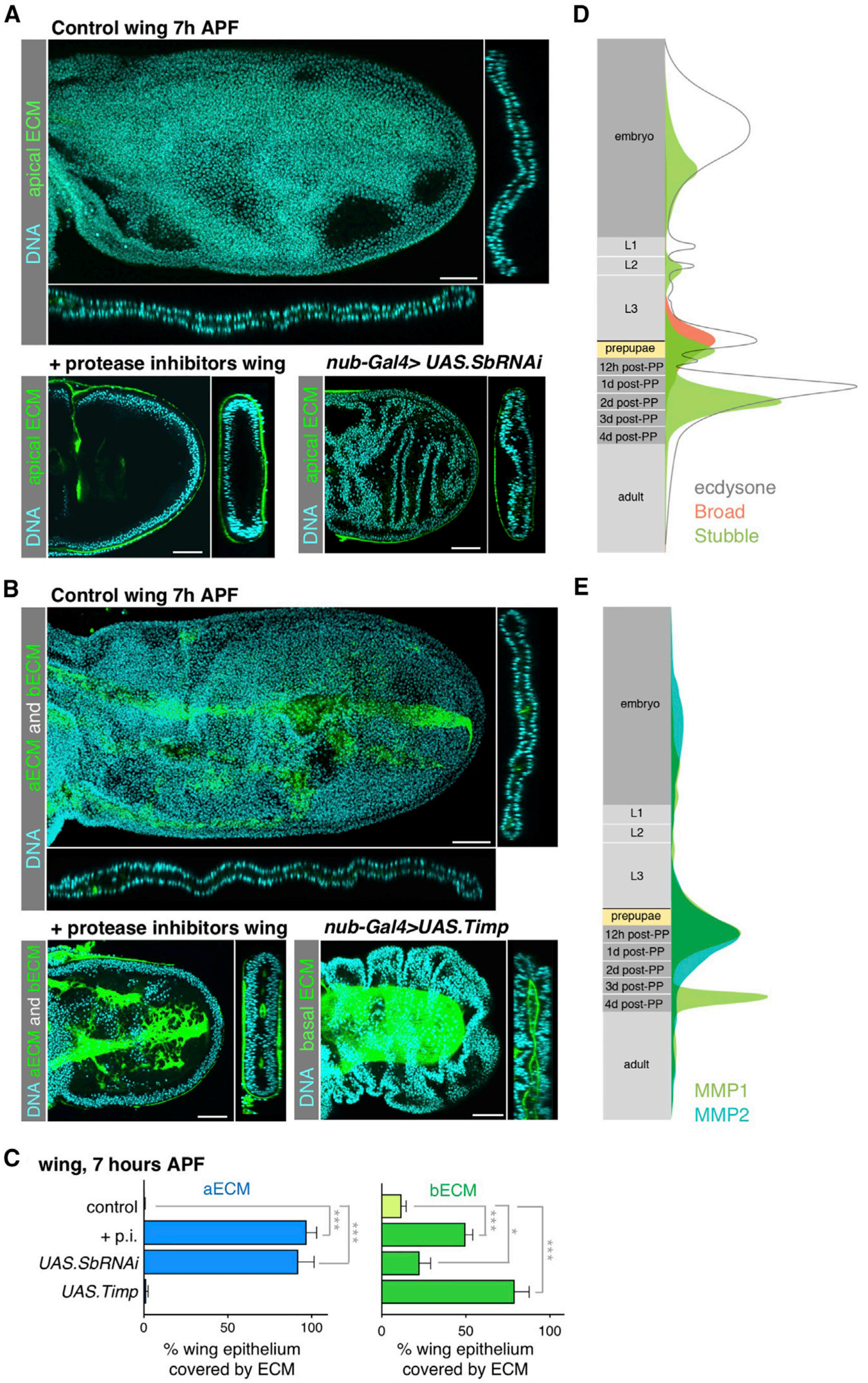

Figure 2. Preventing Matrix Degradation Is Sufficient to Block Elongation of the Wing

(A) Cross-sections of 7 hr APF control (*dp-YFP/+*) (top), *dp-YFP/+* wings treated *ex vivo* with a protease inhibitor mix (bottom left), or Stubble protease RNAi *dp-YFP/+* wings (bottom right). Control wings treated with protease inhibitors and Stubble RNAi wings have not expanded and elongated at 7 hr APF, and are still covered by apical Dp-YFP even though the peripodial membrane has been released. Scale bars, 50 μm.

(B) Cross-sections of 7 hr APF control (*dp-YFP nub-Gal4/vkg-GFP*) wings (top), control wings treated with protease inhibitors (bottom left), and *Timp*-overexpressing wings (*vkg-GFP nub-Gal4>UAS.Timp*) (bottom right). Wings treated with protease inhibitors and *Timp*-expressing wings fail to expand and elongate after the release of the peripodial membrane. Scale bars, 50 μm.

(C) Quantification of the percentage of wing epithelium covered with apical Dp-YFP and basal Vkg-GFP in protease inhibitors treated (+p.i.), *nub-Gal4>UAS.SbRNAi*, and *nub-Gal4>UAS.Timp* wings. *Timp* overexpression has no effect in Dumpy degradation, and the moderate increase in Collagen IV covering the epithelial basal surface under Stubble inhibition compared with control wings is likely due to the decrease in wing surface. Average and SD are presented; n > 4 for each developmental stage. Statistically significant differences between the control and the experimental conditions are indicated (*$p < 0.05$, ***$p < 0.001$).

(D) Schematic graph showing the timing of Stubble protease mRNA induction just following the peak of ecdysone synthesis, and expression of the Broad transcription factor mRNA at pupariation (adapted from www.flybase.org, modEnCODE Development RNA-Seq database; Graveley et al., 2011; modENCODE Consortium et al., 2010).

(E) Schematic graph showing the timing of MMP1/2 protease mRNA induction at pupariation (adapted from www.flybase.org, modEnCODE Development RNA-Seq database; Graveley et al., 2011; modENCODE Consortium et al., 2010).

See also Figure S1.

starts metamorphosis, initiating the transformation of the imaginal disc into the adult appendages (Riddiford, 1993). The transcription factor Broad is one of the early genes that controls the response to ecdysone, and is essential for the elongation of *Drosophila* appendages at that stage (Kiss et al., 1988). Since defects in appendage morphogenesis in *broad* mutants are enhanced when combined with mutations in *Stubble* (Beaton et al., 1988; Kiss et al., 1988), it is likely that Broad controls wing elongation at least in part by inducing degradation of the apical ECM via Stubble. Similar to *broad* and *Stubble*, *MMP1* expression increases in the wing from 0 to 6 hr APF (Guo et al., 2016; Ward et al., 2003; Appel et al., 1993), and a peak in *broad* expression precedes a strong increase in the two types of proteases, apical Stubble, and basal MMPs (Figures 2D and 2E), suggesting that MMPs could be regulated in a similar way to the Stubble protease. These observations point to the expression of apical and basal ECM degrading enzymes as a key part of the response to ecdysone that mediates wing elongation during metamorphosis.

Wing Shape Change Occurs through Early Convergent Extension and Late Isotropic Expansion

Although the columnar-to-cuboidal transition drives wing surface expansion, it does not explain how the wing also elongates

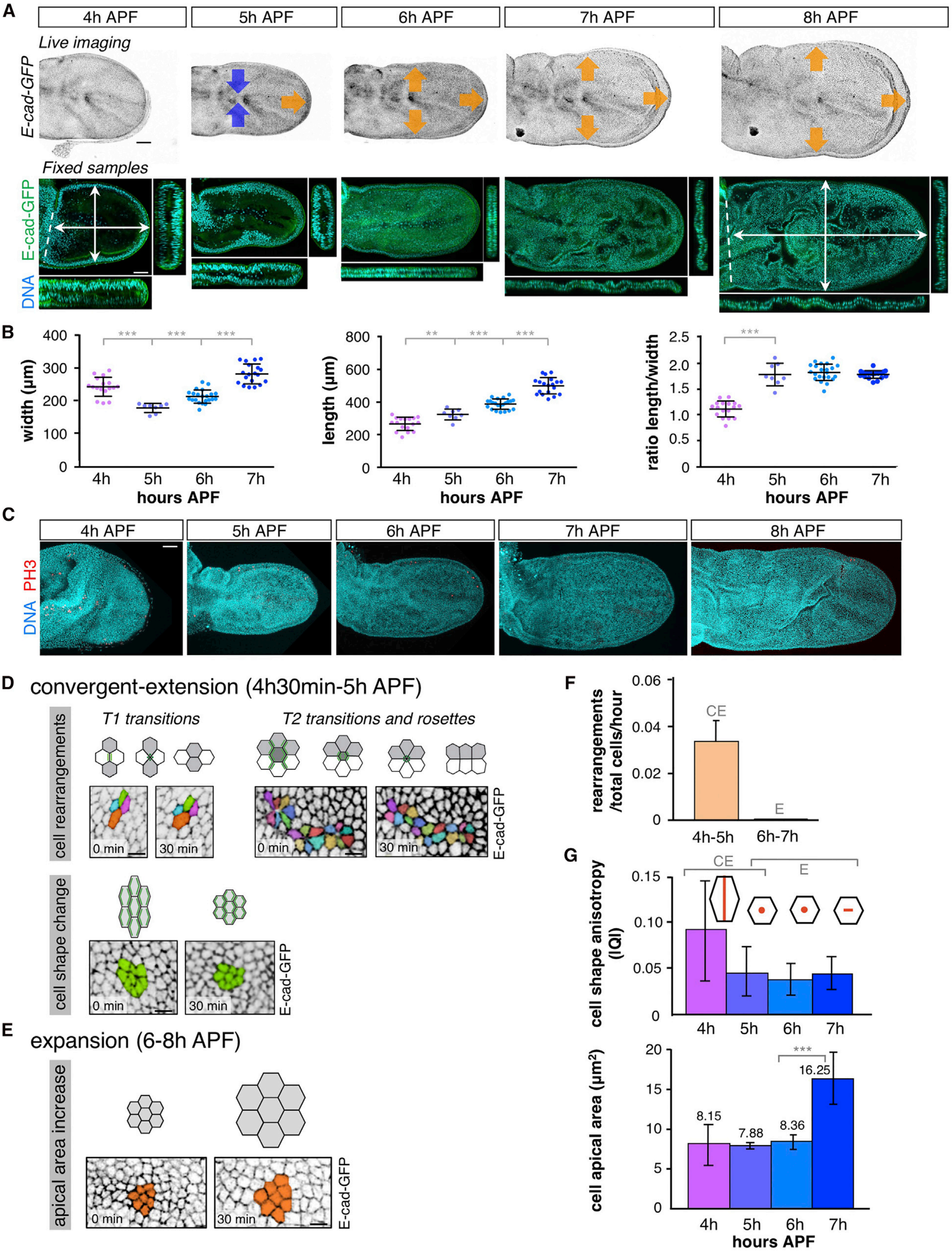

(legend on next page)

along the proximal-distal (PD) axis. We therefore observed the process of wing morphogenesis in both live imaging and fixed samples, which reveal that elongation occurs during the initial stage of wing expansion, while the late stages of wing expansion are nearly isotropic (Figure 3A and Video S1). Quantification of wing width and length over time reveals convergence (a decrease in width) and extension (an increase in length) occurring between 4 and 5 hr APF, following which both width and length increase isotropically (Figure 3B). Notably, oriented cell divisions cannot account for the elongation of the wing, because few if any mitotic cells are observed between 4 and 7 hr APF (Figure 3C). These results confirm that the wing elongates via convergent extension movements that occur prior to the process of isotropic wing expansion.

Live imaging of the early wing-extension phase reveals cell shape changes, and cell intercalation events in which cells rearrange via classic "T1" neighbor exchange, "T2" extrusion, and "rosette" formation (Blankenship et al., 2006) (Figures 3D–3F and S2A; Videos S2–S4). Live imaging of the later wing-expansion phase reveals a progressive isometric increase in apical cell area and wing surface area as cells become more cuboidal (Figures 3E and 3F; Video S5). To analyze the contribution of cell shape and apical area to changes in wing shape and size during convergent extension and expansion, we performed segmentation of the apical surface of cells within wing fixed samples from 4 to 7 hr APF, using E-cad-GFP to detect apical cell membranes (Figures 3G and S2). We found that the relative increase in apical cell area is nearly equal to the relative increase in wing area, indicating that the tissue area expansion is driven by cell area increase (Figures S2D–S2F). Both cell and wing area dramatically increased at the end of the process, from 6 to 7 hr APF. We then quantified the average cell elongation in the wing using a nematic tensor of cell elongation, obtained from a triangulation of the network of cellular junctions (Etournay et al., 2015). We found that before peripodial membrane release, cells in the wing disc are elongated in the anterior-posterior (AP) direction (Figure S2E). During convergent extension the cell shape anisotropy decreases, resulting in roughly isotropic cells. During expansion, the anisotropy of both cell shape and wing shape remains approximately constant (Figures S2D and S2E). We then asked whether changes in cell shape anisotropy account for tissue convergent extension, by comparing the rate of cell elongation with the rate of tissue elongation (Figure S2G). In the absence of tissue deformation arising from cellular rearrangements, these quantities should be equal (Popovic et al., 2017; Etournay et al., 2015), but we found instead that tissue elongation occurs at a faster rate than cell elongation, indicating that cellular rearrangements do contribute to wing elongation. The maximum rate of shear growth due to cell rearrangements is ~0.17 per hour, higher than the one observed in high-resolution live-imaging experiments (compare Figures 3F and S2G). One possibility is that imaging conditions were associated with increased phototoxicity to the tissue, causing the wings to elongate less well than that observed in lower-resolution videos or in fixed samples (compare Figure 3A and Video S1 with Video S6).

Overall, our data show that the increase in wing anisotropy during convergent extension relies on both the change in cell shape and the cumulative effect of cell intercalations, while the increase in wing area during expansion is entirely accounted for by the increase in cell area. Convergent extension is thought to be brought about by planar polarized junctional tension (Blankenship et al., 2006; Bertet et al., 2004), a point supported by computer simulations in two dimensions (Lan et al., 2015; Rauzi et al., 2008). We therefore sought to identify possible planar polarizing mechanisms that can drive tissue elongation.

Dynamic Changes in the Localization of Myosin II Drive Convergent Extension and Expansion

An important polarizing mechanism for convergent extension is the planar polarization of Myosin-II, first discovered in the *Drosophila* embryo (Bertet et al., 2004; Zallen and Wieschaus, 2004). We analyzed the distribution of the Myosin-II regulatory light chain (encoded by the *spaghetti squash* or *sqh* gene in *Drosophila*) using a GFP-tagged transgene expressed from the endogenous promoter in a genetic background mutant for the endogenous gene (sqh^{AX3}; {*sqh-GFP*}). We found that Myosin-II is planar polarized during convergent extension between 4 and 5 hr APF (2-fold increase in Myosin-II fluorescence intensity along the PD axis compared with the AP axis), and that it later relocalizes from the apical to the lateral sides of the cell as the tissue expands isotropically from 6 to 8 hr APF (Figures 4A, 4B, S3A, and S3B). Thus, initial planar polarization of Myosin-II

Figure 3. The Wing Elongates by Convergent Extension Followed by Isotropic Expansion

(A) E-cad-GFP live imaging (top) and fixed imaging (bottom) of developing wings from 4 to 8 hr APF. From 4 to 5 hr APF, the wing contracts along the anterior-posterior (blue arrows) axis and elongates along the proximal-distal axis (orange arrow) consistent with convergent extension. From 6 to 8 hr APF, the wing expands isotropically in all directions (orange arrows). Scale bar, 50 μm. See also Video S1.

(B) Quantification of the maximal width and length of fixed samples from 4 to 7 hr APF. Wing anisotropy is established from 4 to 5 hr APF, through width decrease and length increase. From 6 to 7 hr APF both parameters increase at the same rate, maintaining a constant ratio. Average, SD, and individual data points are presented; n > 8 for each developmental stage. Statistically significant differences are indicated (**$p < 0.005$, ***$p < 0.001$).

(C) Cell division (phospho-histone-H3-positive nuclei, red) is very rare in the developing wing from 4 to 8 hr APF. Scale bar, 50 μm.

(D and E) E-cad-GFP live imaging during convergent extension (D, see Videos S2–S4) and expansion (E, see Video S5). Cell tracking (colored cells) shows how the epithelia contract along the anterior-posterior axis during convergent extension by cell rearrangements (T1, T2 transitions and rosettes) and cell shape changes, whereas they expand isotropically during expansion. Scale bar, 10 μm.

(F) Quantification of cell rearrangements detected in high-space-resolution live-imaging experiments during convergent extension (CE) and expansion (E) in *E-cad-GFP*-expressing wings (n = 4 wings, >1,500 cells per wing). Cell rearrangements only take place during convergent extension. Average and SD are presented.

(G) Quantification of cell shape anisotropy and apical surface area of epithelial cells from 4 to 7 hr APF. Data were obtained by segmentation of fixed pupal wings expressing *E-cad-GFP* (n = 13 wings, >5,000 cells per wing). Cell anisotropy decreases while apical area remains constant during convergent extension. During expansion, cells maintain their isotropy and apical surface area increases. Average and SD are presented, and statistically significant differences are indicated (***$p < 0.001$).

See also Figure S2.

CellPress

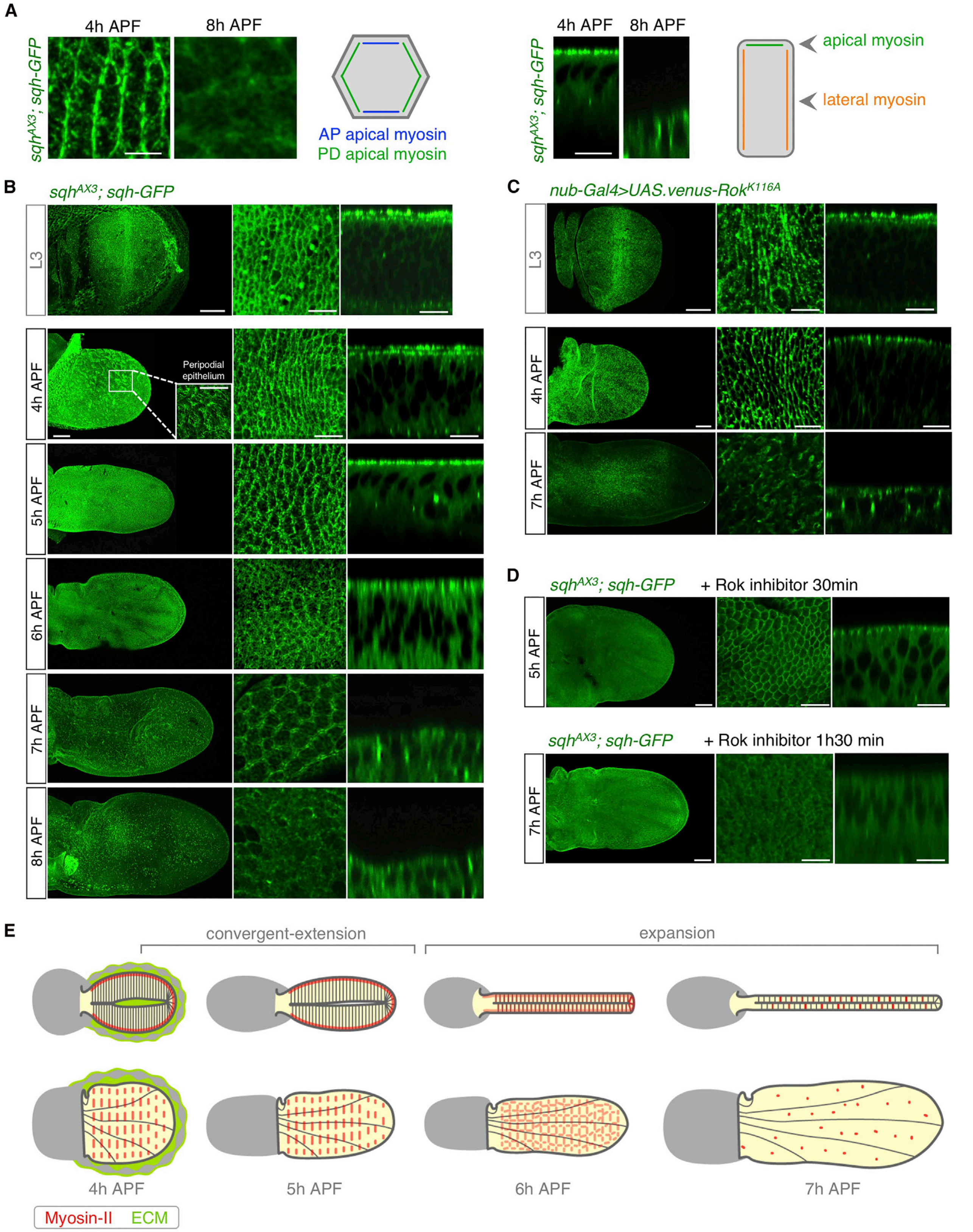

(legend on next page)

correlates with initial anisotropic convergent extension, and subsequent lateral relocalization of Myosin-II correlates with isotropic tissue expansion as cell height decreases.

Rho-kinase (Rok) is responsible for activating Myosin-II contractility by directly phosphorylating the Myosin-II regulatory light chain. We found that Rok localized in precisely the same fashion as Myosin-II in third instar larval wing discs and in the early pupal wing at 4 hr APF and also dissipated apically by 7 hr APF (Figure 4C). Rok activity is required for convergent extension and expansion of the wing, since the addition of the Rok inhibitor Y-27632 to cultured wings completely abolished the elongation of the wing between 4 hr 30 min and 5 hr APF and wing expansion between 5 hr 30 min and 7 hr APF in culture (Figure 4D and Video S7). By adjusting parameters in a continuum model (Popovic et al., 2017) to experimental data of cell and tissue elongation, we found that Myosin-II polarization can account for anisotropic cell shape changes and cellular rearrangements during wing elongation (Figure S3). These results define an essential role for planar polarized Rok and Myosin-II in driving axial convergent extension to elongate the tissue.

Classical Planar Polarization Systems Are Not Required for Wing Convergent Extension

We next considered how Myosin-II undergoes dynamic changes in its localization during wing development. We considered two hypotheses for the initial planar polarization of Myosin-II leading to convergent extension: (1) a developmentally programmed pattern of gene expression that orients a planar polarity system or (2) a developmentally programmed pattern of tissue growth that generates global tensile forces within the constraining environment of the ECM.

We found that none of the known planar polarity systems, Dachsous-Fat cadherin, Frizzled, or Par3/Bazooka (Baz), affects the anisotropic growth of the wing. *fat* mutant wings complete the process of expansion and elongation normally during pupal development, although they are rounded prior to expansion and elongation (Figure S4A). Mutants in *frizzled* or genes encoding other pathway components of the Frizzled planar polarity system, such as *flamingo*/*starry night* or *van gogh*/*strabismus*, do not affect limb elongation in *Drosophila* (Chae et al., 1999; Lu et al., 1999; Taylor et al., 1998; Wolff and Rubin, 1998). Similarly, depletion of Baz, which is necessary for embryo germband extension (Zallen and Wieschaus, 2004), does not affect wing elongation, and is not planar polarized in the elongating wing (Figures S4B and S4C), where it instead localizes to adherens junctions (Figure S4D). Thus, *Drosophila* wing convergent extension, mediated by Myosin-II polarization, does not depend on any known planar polarity system.

Myosin II planar polarization could conceivably result from the circumferential stretch pattern induced by a differential cell proliferation rate along the future PD axis, which is sufficient to planar polarize Myosin-II orthogonal to the PD axis by the third larval instar stage (Figure 4B) (Legoff et al., 2013; Mao et al., 2013). Myosin-II remains polarized during disc eversion between the third larval instar and 4 hr APF, at which point the ECM is released and Myosin-II is able to produce convergent extension movements.

We next considered how Myosin-II relocalizes from the apical ring of adherens junctions to lateral membranes to drive wing expansion from 6 to 8 hr APF. We noticed that GFP-tagged Rok also relocalizes to lateral membranes, as do the adherens junctions themselves, as marked by β-Catenin/Armadillo (Arm) (Figures 4C and S4E). Myosin-II localizes adjacent to adherens junctions in the lateral membrane at 7 hr APF (Figure S4D). This result suggests that relocalization of Rok, Myosin-II and adherens junctions might be linked events. One potential mechanism for repositioning adherens junctions involves the Par-3/Baz protein, shown to be important for junctional movement laterally in *Drosophila* embryos (Wang et al., 2012). However, we found that Baz does not relocalize laterally with Rok/Myosin-II or Arm during the phase of wing expansion at 7 hr APF (Figures S4C–S4E). We favor the simplest model for wing expansion, which is that after removal of the ECM the columnar cells begin to return to their more energetically favorable cuboidal form, gradually expanding the apical surface and dissipating the entire apical actomyosin ring, which is known to be tension dependent (Lecuit and Yap, 2015; Fernandez-Gonzalez et al., 2009). In the absence of an apical actomyosin ring, adherens junctions are no longer restricted apically and can spread

Figure 4. Dynamic Changes in the Localization of Myosin II Drive Convergent Extension and Expansion

(A) Apical wing cross-sections at 4 and 8 hr APF (left). At 4 hr APF Myosin II-GFP is planar polarized along the proximal-distal axis, whereas at 8 hr APF is homogenously distributed (see Figure S3A). Lateral wing cross-sections at 4 hr APF and 8 hr APF (right). From 4 to 8 hr APF, Myosin II-GFP relocates from the apical surface to the lateral membranes (see Figure S3B). Scale bar, 10 μm.

(B) Myosin II-GFP localization in developing *Drosophila* wings. Maximum projections (left), high-magnification apical-view xy sections (middle), and lateral-view z sections (right) of *sqhAX3*; {*sqh-GFP*} wings are shown. Myosin-II is already polarized along the proximal-distal axis at third instar larval wing discs (L3). During convergent extension Myosin-II-GFP (encoded by *sqh-GFP*, green) accumulates in apical cables, polarized to drive contraction along the anterior-posterior axis. At 6 hr APF, Myosin-II-GFP starts to relocate the lateral side of the cells, and is strongly localized at the lateral domain from 7 to 8 hr APF. Inset at 4 hr APF shows a high-magnification view of the peripodial membrane, which is subsequently removed. Scale bars, 50 μm (left) and 10 μm (middle and right).

(C) Similarly to Myosin-II, Rok-GFP is planar polarized in the imaginal wing disc at late larval stages, but localizes laterally during the expansion phase. Scale bars, 50 μm (left) and 10 μm (middle and right).

(D) Treatment of cultured wings with the Rho-kinase inhibitor Y-27632 for 30 min impairs convergence extension and decreases planar polarization of Myosin-II-GFP in 4 hr 30 min APF wings (see Video S7), and cell flattening and wing extension is impaired in 8 hr 30 min APF wings after 1 hr 30 min treatment. Scale bars, 50 μm (left) and 10 μm (middle and right).

(E) Schematic diagram of wing disc morphogenesis from 4 to 7 hr APF, in apico-basal (top) and sagittal (bottom) cross-sections. Wing blade is shown in yellow and peripodial membrane in gray. Apical and basal ECM are shown in green, and Myosin-II in red. During convergent extension (from 4 hr to 8 hr APF), Myosin-II is localized apically, planar polarized along the anterior-posterior axis to mediate the elongation of the tissue. During expansion, from 6 hr APF, Myosin-II relocalizes to the lateral membrane, concomitant with the isotropic expansion of the wing and the flattening of the columnar epithelia to a cuboidal one.

See also Figures S3 and S4.

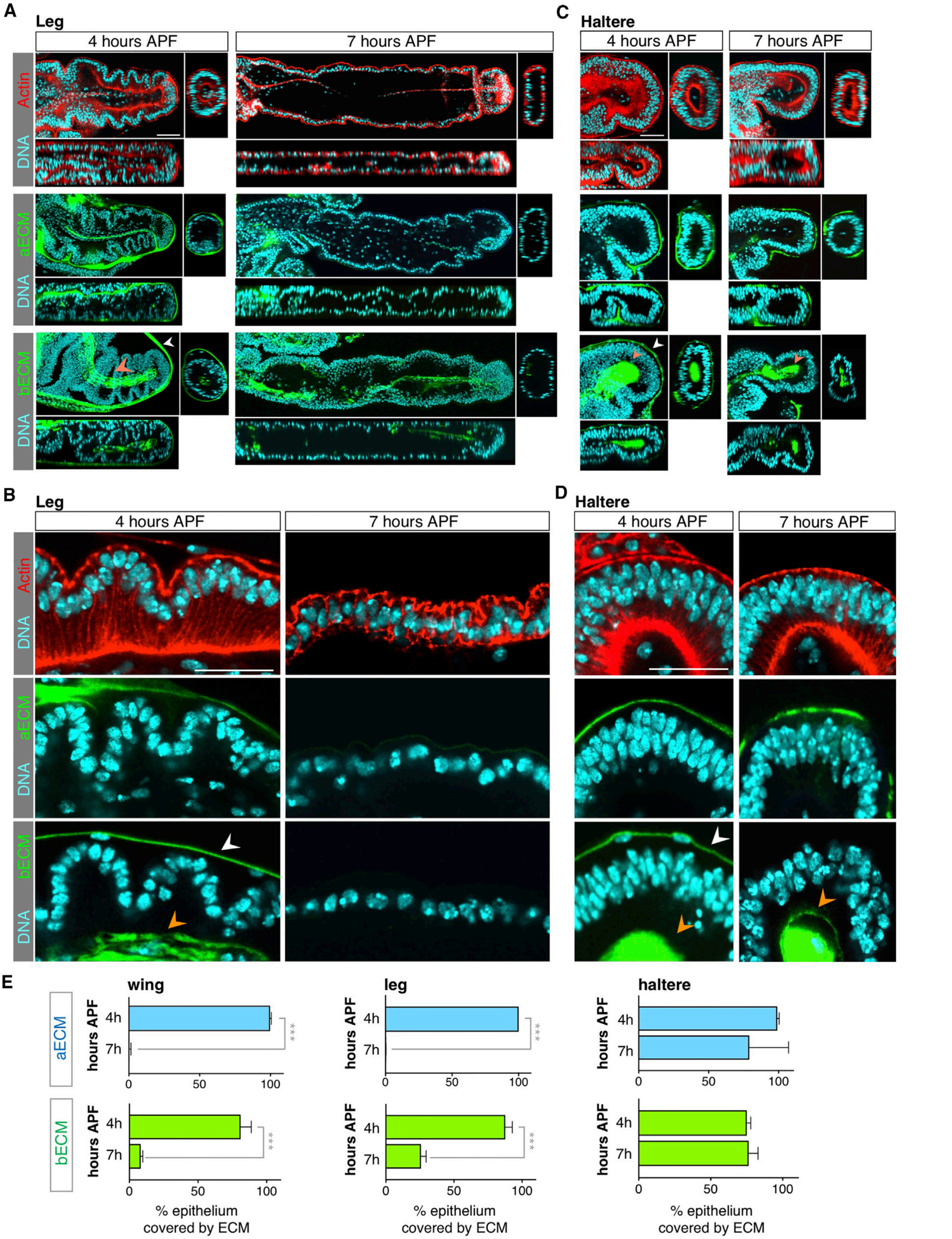

(legend on next page)

along the entire lateral membrane, taking some remaining clusters of contractile actomyosin (Rok/Myosin-II) with it (Figures 4A–4C and 4E). The lateral actomyosin clusters then contribute actively to cell shape change from columnar to cuboidal, since Rok-inhibitor treatment prevents transformation to a cuboidal cell shape by 7 hr APF (Figure 4D).

Tissue-Specific Control of Matrix Remodeling by the Hox Gene Ultrabithorax

To confirm the generality of our findings in the wing, we examined whether the same mechanism operates during elongation of the leg. Once again, we find that both apical and basal matrices remain in place while leg cells are still columnar at or before 4 hr APF, but that the matrix begins to be degraded at 5 hr APF as the leg expands and extends (Figures 5A, 5B and 5E). These results confirm that matrix remodeling immediately precedes morphogenetic change in both the wings and legs of *Drosophila*. One limb of *Drosophila* that famously fails to extend is the haltere, a tissue that was once a wing in the four-winged ancestors of *Drosophila*, but that evolved into a vestigial stump upon the evolutionary selection for two-winged insects (order Diptera). We therefore characterized the ECM distribution and cell shapes of the haltere at 4–7 hr APF. We found that the haltere is initially composed of pseudo-stratified columnar cells at 4 hr APF and strikingly remains so all the way through to 7 hr APF, such that the haltere does not flatten or extend (Figures 5C–5E). Notably, the ECM remains present at both the apical and basal surface of the haltere throughout this process, despite the removal of the overlying peripodial layer. To confirm our findings with Collagen IV, we examined GFP-tagged forms of two other basal ECM components, Laminin (Laminin β1-subunit encoded by *LanB1*, Lanβ1-GFP), and Perlecan (Pcan-GFP). We found that, similar to Collagen IV, both are degraded between 4 and 7 hr APF in the wing and leg, but not in the haltere (Figures 6A–6G and S5).

To test whether degradation of the ECM is sufficient to allow the haltere to flatten and extend, we added the serine protease enzyme trypsin to cultured halteres. We found that addition of this enzyme for 15 min allowed the haltere to flatten and induced loss of both the apical and basal matrix (Figures 7A and 7B). The result is a "winglet"-like structure that is much smaller than the wing itself at this stage due to the fact that the haltere comprises fewer cells than the wing throughout its early growth phase. These data show that inducing degradation of the ECM can drive flattening and expansion of the haltere epithelia, although we cannot rule out that degradation of additional proteins by trypsin could contribute to change cell shape. To examine why matrix degradation fails to occur in the haltere, which like the wing and leg expresses both *broad* and *MMP1* (Figure S1), we considered the role of the Hox transcription factor Ultrabithorax (Ubx), a master control gene governing the switch between wing and haltere development whose loss-of-function mutation produces four-winged *Drosophila* (Pavlopoulos and Akam, 2011; Lewis, 1978).

To test whether Ubx controls the decision not to degrade the ECM in the haltere, we inactivated Ubx after the growth phase by expressing RNAi against Ubx in late larval imaginal discs with the *Gal4/UAS* conditional expression system. Loss of Ubx in the pupal stages resulted in degradation of both apical and basal ECM, allowing the flattening of the pseudo-stratified columnar epithelium to a cuboidal one, which expands and extends the haltere into a "winglet" (Figures 7B and 7C). Thus, Ubx is specifically required for restricting haltere morphogenetic expansion and elongation, independently of its known roles in restricting haltere growth and patterning. We next tested the gain-of-function phenotype of *Ubx* expression in the wing, which has been reported to cause transformation into a haltere-like structure but whose mechanism of action has remained unclear (Pavlopoulos and Akam, 2011). We find that overexpression of Ubx is sufficient to prevent apical and basal matrix degradation in the wing such that the wing fails to expand and extend by 7 hr APF, resulting in a tiny adult structure that resembles the haltere (Figures 7D and 7E). This phenotype is similar to that caused by culturing the wing discs *ex vivo* in the presence of a protease inhibitor cocktail, such that the tissue fails to elongate at 7 hr APF (Figure 2). Thus, matrix remodeling can be controlled in a tissue-specific fashion by a master developmental control gene, whose increased expression at the beginning of metamorphosis (Figures 7F and S1) blocks matrix remodeling to prevent columnar-to-cuboidal shape change and tissue morphogenesis.

Comparison of Myosin-II Localization Dynamics in the Wing, Leg, and Haltere

We next asked whether the dynamic changes in Myosin-II localization we observe during elongation of the wing also occur during elongation of the leg. We examined Myosin-II-GFP and

Figure 5. Remodeling of the Extracellular Matrix Also Occurs in the Leg, but Not the Haltere

(A) *Drosophila* leg cross-sections at 4 hr APF, before peripodial membrane release, and 7 hr APF, after anisotropic expansion and tissue flattening. As in the developing wing, elongation of the leg involves degradation of apical Dp-YFP and basal Vkg-GFP in contact with the leg epithelial cells (orange arrowhead) just prior to tissue elongation, which proceeds by columnar-to-cuboidal cell shape change. At 4 hr APF a layer of basal Vkg-GFP covers the basal surface of the peripodial membrane cells (white arrowhead). Scale bar, 50 μm.

(B) High-magnification view of epithelial cells of the leg disc at 4 hr APF, in contact with apical Dp-YFP and basal Vkg-GFP (orange arrow), and at 7 hr APF, when the matrix is removed and cells adopt a cuboidal-like morphology. At 4 hr APF epithelial cells are columnar and all reach the basal surface, as shown by the actin cytoskeleton (red), and a layer of basal Vkg-GFP covers the basal surface of the peripodial membrane cells (white arrowhead). Scale bar, 25 μm.

(C) Developing haltere cross-sections at 4 hr APF, before peripodial membrane release, and 7 hr APF, after peripodial membrane release. In contrast with wing and leg development, the haltere disc does not extend from 4 to 7 hr APF, and apical Dp-YFP and basal Vkg-GFP (orange arrowhead) cover the haltere epithelia during the entire process. The ECM remains present at both the apical and basal surface of the haltere throughout this process, despite the removal of the overlying peripodial layer. The layer of basal Vkg-GFP that covers the basal surface is only detectable at 4 hr APF (white arrowhead). Scale bar, 50 μm.

(D) High-magnification cross-section of pseudo-stratified columnar haltere epithelia at 4 and 7 hr APF in contact with apical Dp-YFP and basal Vkg-GFP (orange arrowheads). At 4 hr APF Vkg-GFP also covers the basal surface of the peripodial membrane (white arrowhead). Scale bar, 25 μm.

(E) Quantification of the percentage of epithelium covered with apical Dp-YFP and basal Vkg-GFP in control wings, legs, and halteres at 4 and 7 hr APF. Average, SD, and individual data points are presented; $n > 4$ for each developmental stage. Statistically significant differences are indicated (***$p < 0.001$).

CellPress

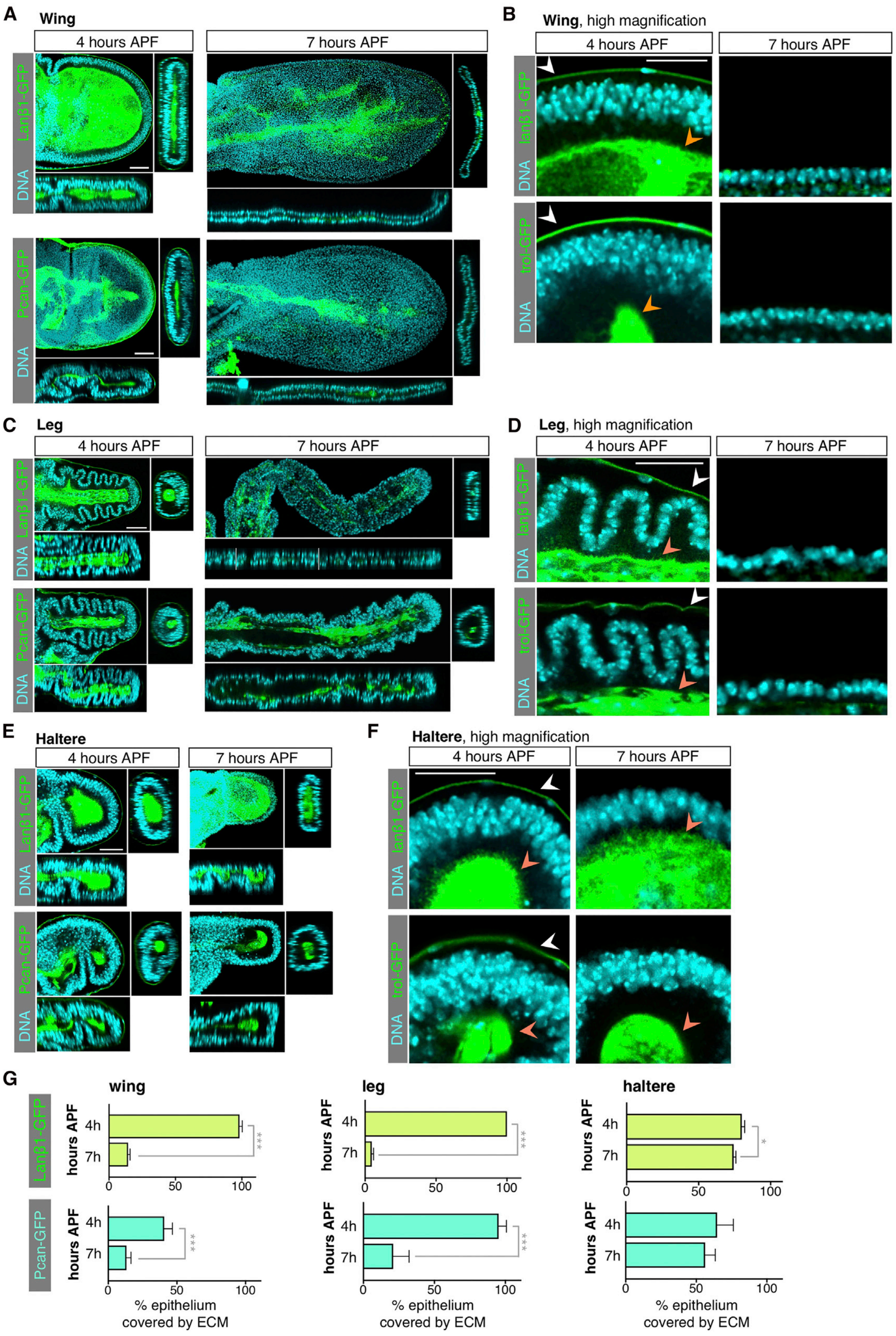

(legend on next page)

Rok-Venus localization in the larval leg imaginal disc and in pupal legs at 4–7 hr APF (Figures S6A–S6C). We find that, as in the wing, planar polarization of Myosin-II and Rok begins in the larval stages in a circumferential pattern around the central region of the leg disc. After eversion, this centralmost region becomes the distal-most point of the elongating leg. Upon leg elongation, the planar polarization of Myosin-II dissipates, and Myosin-II then relocalizes laterally and then basally as cells undergo columnar-to-cuboidal transition, again similar to the wing. Differences between the wing and leg include the overall flat blade versus round tube form, and the more extensive folding of the leg while still encapsulated within the ECM, which appears to involve early apical matrix degradation and PD elongation preceding release of the basal matrix (Figure S7). This early PD elongation of the apical surface and consequent folding provides an explanation for why Myosin-II planar polarization dissipates more rapidly in the leg (Figures S6A–S6C). Notably a similar folded morphology is induced in the wing upon expression of *Timp* to prevent basal matrix degradation, which is rescued when Dumpy degradation is inhibited by adding the protease inhibitor mix, supporting the view that folding arises from elongation of the apical surface while the basal surface remains attached to the matrix (Figure S7).

We also examined the localization of Myosin-II in the haltere, which remains encapsulated within the ECM. Firstly, the initial planar polarization of Myosin-II is much weaker in the haltere than the wing at the same stage, presumably due to the much-reduced growth of the haltere compared with the wing (Figures S6D–S6F). Secondly, unlike the wing and leg, Myosin-II does not relocalize laterally or basally by 7 hr APF in the haltere. These findings support the notion that matrix remodeling in the wing and leg is the trigger than induces subsequent dynamic changes in Myosin-II localization to drive morphogenetic elongation.

DISCUSSION

Our results show that morphogenetic elongation of *Drosophila* limbs occurs via two processes: convergent extension and expansion. Convergent extension is driven by planar polarized localization of Myosin-II, which drives both anisotropic cell shape change and intercalation of cells such that the tissue contracts along the AP axis and extends along the PD axis. Expansion involves relocalization of Myosin-II from the apical ring to lateral membranes, which promotes columnar-to-cuboidal transition and thus isotropic tissue expansion. These processes are sequentially induced following release of the constraining force of the ECM. Remodeling of the matrix is subject to at least two distinct forms of developmental control. First there is temporal control of matrix removal, which is timed to coincide with removal of the peripodial membrane, and is mediated by hormonal signals including ecdysone, which induces the apical matrix protease Stubble and the basal matrix proteases MMP1 and MMP2 (Figures 2D, 2E, and S1). The transcription factor Broad is induced by ecdysone at pupariation (Figures 2D and S1) and is likely to mediate induction of protease expression as *broad* mutants fails to undergo limb elongation (Karim et al., 1993; Beaton et al., 1988; Kiss et al., 1988; Mandaron, 1970). Second, there is tissue-specific control of matrix removal, which occurs in the wings and legs but not in the halteres (despite simultaneous removal of the peripodial layer from all three limbs) due to the haltere-specific master control gene *Ubx* (Figures 7F and S1) (Lewis, 1978).

Placing these results in the context of the earlier stages of limb development, we note that both the apical and basal ECM components are present as limbs grow during the larval "imaginal disc" stages of life (Ray et al., 2015; Pastor-Pareja and Xu, 2011). We propose that the matrix provides an elastic constraining force along the apical and basal surface of the epithelium, but not along the lateral sides of each cell. This constraining force pattern promotes columnar epithelial cell shape, because the lateral sides are able to grow longer than the apical or basal sides of each cell as the tissue increases in mass (Pastor-Pareja and Xu, 2011). Our data show that release of this constraining force by developmentally controlled matrix remodeling allows each epithelial cell to return to a more cuboidal form, which involves shrinkage of the lateral sides and expansion of the apical and basal sides to expand the entire tissue.

In the presence of a chitinous exoskeleton in late wing development, Dumpy has an essential role as a mediator of the epidermal-cuticle junction (Etournay et al., 2015; Ray et al.,

Figure 6. Remodeling of Basal Extracellular Matrix Laminin and Perlecan Occurs during Wing and Leg Elongation but Does Not Happen in the Haltere

(A) Cross-sections of developing wings at 4 and 7 hr APF expressing the basal ECM components Laminin-GFP (Laminin β1-subunit encoded by *LanB1*, Lanβ1-GFP) and Perlecan-GFP (Pcan-GFP). At 4 hr APF, Lanβ1-GFP and Pcan-GFP cover the basal surface of the wing epithelia and surround the basal side of the peripodial membrane. At 7 hr APF the matrix has been degraded and only some traces are still detected. Scale bars, 50 μm.

(B) High-magnification view of epithelial cells showing that, at 4 hr APF, columnar epithelial cells are in contact with basal Lanβ1-GFP and Pcan-GFP (orange arrowheads), whereas by 7 hr APF the matrix is absent. A layer of basal ECM covers the basal surface of the peripodial membrane cells at 4 hr APF (white arrowheads). Scale bar, 25 μm.

(C) *Drosophila* leg cross-sections at 4 hr APF, before peripodial membrane release, and 7 hr APF. Elongation of the leg involves degradation of the basal ECM components Lanβ1-GFP and Pcan-GFP prior to tissue elongation. Scale bar, 50 μm.

(D) High-magnification view of epithelial cells of the leg disc at 4 hr APF, in which the basal ECM covers the basal side of the epithelial leg cells (orange arrowheads) and the basal surface of the peripodial membrane (white arrowheads), and at 7 hr APF, when the ECM has been degraded. Scale bar, 25 μm.

(E) Developing haltere cross-sections at 4 hr APF, before peripodial membrane release, and 7 hr APF, after peripodial membrane release. In contrast to wing and leg development, the haltere disc does not extend from 4 to 7 hr APF, and Lanβ1-GFP and Pcan-GFP cover the haltere epithelia during the process. Scale bar, 50 μm.

(F) High-magnification cross-section of pseudo-stratified columnar haltere epithelia at 4 and 7 hr APF in contact with the basal components Lanβ1-GFP and Pcan-GFP (orange arrowheads). The layer of basal ECM that covers the basal surface of the peripodial membrane is only detectable at 4 hr APF (white arrowheads). Scale bar, 25 μm.

(G) Quantification of the percentage of epithelium covered with basal Lanβ1-GFP and Pcan-GFP in control wings, legs, and halteres at 4 and 7 hr APF. Average, SD, and individual data points are presented; n > 4 for each developmental stage. Statistically significant differences are indicated (*$p < 0.05$, ***$p < 0.001$). See also Figure S5.

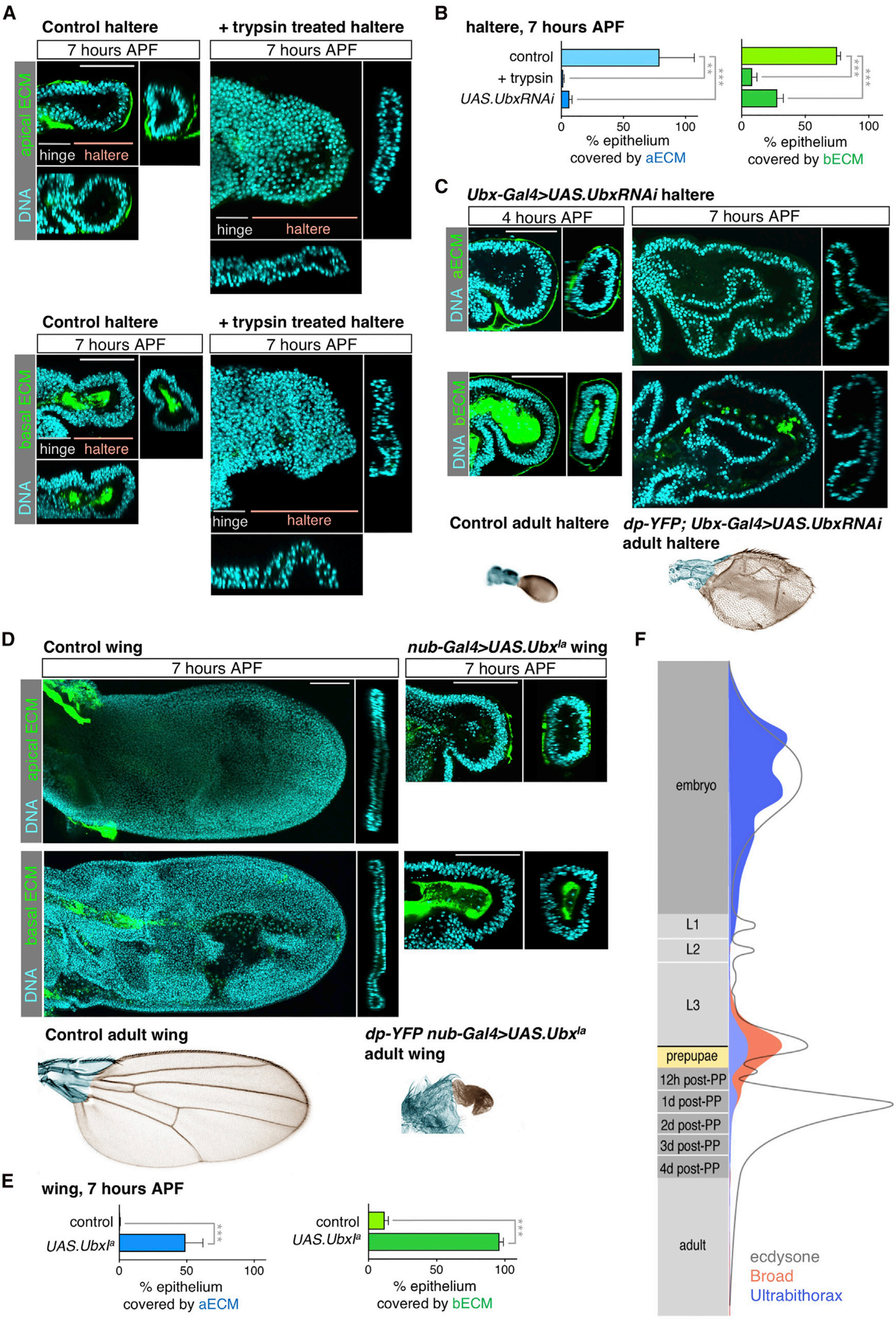

(legend on next page)

2015). During early pupal wing convergent extension and expansion, however, the cuticle has not yet been secreted, which indicates that Dumpy may act instead as a component of the apical ECM itself, a role supported by the structure of Dumpy protein. Each Dumpy molecule is a gigantic structure anchored to the cell membrane at its carboxy-terminal end and arranged into fibers by its epidermal growth factor modules, with less organized regions that confer elasticity. Also, its ZP domain could crosslink different Dumpy molecules with other ECM components (Wilkin et al., 2000). Dumpy's size, physical properties, and capacity to polymerize could build an apical ECM capable of redistributing and resisting tension, providing mechanical strength during morphogenesis of the wing from larval stages.

At the apical surface of the epithelium, differential growth of the larval imaginal disc tissue within the ECM leads to a global stretch pattern that causes planar polarization of Myosin-II orthogonal to the PD axis (Legoff et al., 2013; Mao et al., 2013). This phenomenon of stretch- induced Myosin-II accumulation has also been observed in the embryo (Fernandez-Gonzalez et al., 2009; Fernandez-Gonzalez and Zallen, 2009). Release of the ECM then allows polarized Myosin-II to contract junctions to drive cell shape changes and cell intercalation events, and thus generate convergent extension of the entire tissue.

This elegant "biological spring" mechanism of accumulating mass within an elastic tensile matrix and then inducing its release to trigger morphogenetic change in three dimensions is a classic example of the internal storage of potential energy that is subsequently released to do work, and represents an important function for matrix remodeling in controlling cell shape and tissue morphogenesis (Bonnans et al., 2014; Rodriguez-Fraticelli and Martin-Belmonte, 2014).

Drawing comparisons with other tissue types and other species, we note that the columnar-to-cuboidal transition in *Drosophila* limbs is a process driven entirely by forces intrinsic to each individual cell: cortical actomyosin contractility that is initially weak at lateral membranes but then strengthens upon relocalization of Myosin-II laterally. In this respect, it contrasts with other examples of tissue flattening that are simply driven by external stretching forces imposed by neighboring tissues. Examples include stretching of the *Drosophila* ovarian follicle cells by the force of the growing egg (Haigo and Bilder, 2011), pulling of the *Drosophila* embryonic ectoderm over the contracting amnioserosa (Solon et al., 2009), spreading of the zebrafish enveloping cell layer over the yolk cell by a contractile ring (Xiong et al., 2014; Behrndt et al., 2012; Solnica-Krezel, 2005), or flattening of the trophectoderm layer by accumulation of fluid inside the mammalian blastocyst. Instead, *Drosophila* limb elongation more closely resembles elongation of the primitive gut (archenteron) of the sea urchins *Strongylocentrotus purpuratus* and *Lytechinus pictus* (Hardin and Cheng, 1986; Ettensohn, 1985) as well as elongation of the tentacles of the sea anemone *N. vectensis* (Fritz et al., 2013).

The process of convergent extension in *Drosophila* limbs appears similar to other examples of convergent extension movements in the *Drosophila* embryo and vertebrate primitive streak and kidney tubules, which are also driven by intrinsic local forces driven by planar polarized Myosin-II (Pare et al., 2014; Lienkamp et al., 2012; Voiculescu et al., 2007; Blankenship et al., 2006; Sepich et al., 2005; Bertet et al., 2004; Zallen and Wieschaus, 2004; Irvine and Wieschaus, 1994). However, global forces can also contribute to convergent extension in *Drosophila* and vertebrate embryos (Collinet et al., 2015; Lye et al., 2015; Campinho et al., 2013; Behrndt et al., 2012; Butler et al., 2009; Keller and Trinkaus, 1987; Keller, 1980). There are no extrinsic pulling forces acting globally to stretch *Drosophila* limbs during the early stages of pupal development investigated here, making limb elongation a bona fide example of local Myosin-II planar polarization being solely responsible for convergent extension. Nevertheless, we and others previously showed that global stretch forces do arise much later in pupal development due to redeposition of ECM for patterned attachment of the limbs to the exoskeleton, which serve as anchor points for shaping the tissue into its precise final form (Etournay et al., 2015; Ray et al., 2015).

STAR★METHODS

Detailed methods are provided in the online version of this paper and include the following:

- KEY RESOURCES TABLE
- CONTACT FOR REAGENT AND RESOURCE SHARING

Figure 7. Ubx Blocks Matrix Degradation to Prevent Elongation of the Haltere

(A) Cross-sections of the developing haltere at 7 hr APF. Degradation of Dp-YFP (top) and Vkg-GFP (bottom) during haltere development by addition of trypsin protease for 15 min results in the elongation and flattening of the haltere, coupled with columnar-to-cuboidal cell shape change. The result is a "winglet"-like structure that is much smaller than the wing itself at this stage due to the fact that the haltere comprises fewer cells than the wing throughout its early growth phase. Haltere and hinge epithelia are highlighted. Note that number of nuclei in the cross-sections is similar in the control and the treated haltere (approximately 40); the apparent increase in cell number in the treated haltere is an effect of the tissue being flattened and expanded. Scale bar, 50 μm.

(B) Quantification of the percentage of epithelium covered with apical Dp-YFP and basal Vkg-GFP in 7 hr APF control, trypsin treated, or expressing *UbxRNAi* (*Ubx-Gal4>UAS.UbxRNAi*) halteres. Average and SD are presented; n > 4. Statistically significant differences are indicated (**$p < 0.005$, ***$p < 0.001$).

(C) Cross-sections of the entire developing haltere at 4 and 7 hr APF in *dp-YFP/+*; *Ubx-Gal4>UAS.UbxRNAi*, and in *vkg-YFP/+*; *Ubx-Gal4>UAS.UbxRNAi* halteres (top). Loss of Ubx resulted in degradation of both apical and basal ECM and consequent expansion and extension of the haltere. Adult halteres from control and animals expressing RNAi against Ubx. Depletion of Ubx transform the haltere into a "winglet" (bottom). Scale bars, 50 μm.

(D) Cross-sections of 7 hr APF control and wings (top) ectopically overexpressing gain-of-function Ubx allele (*nub-Gal4>UAS.Ubx^{la}*), which has been reported to cause transformation into a haltere-like structure, but whose mechanism of action has remained unclear (Pavlopoulos and Akam, 2011). *Ubx*-expressing wings have not expanded and elongated at 7 hr APF, and are still covered by apical *dp-YFP* or Vkg-GFP, even though the peripodial membrane has been released. Bottom: adult wings from control and from animals overexpressing the gain-of-function Ubx allele. Scale bars, 50 μm.

(E) Quantification of the percentage of epithelium covered with apical Dp-YFP and basal Vkg-GFP in 7 hr APF control and overexpressing *Ubx^{la}* wings. Average and SD are presented; n > 4. Statistically significant differences are indicated (***$p < 0.001$).

(F) Schematic graph of the timing of the homeobox gene Ubx mRNA induction just following the peak of ecdysone synthesis at pupariation (adapted from www.flybase.org, modEnCODE Development RNA-Seq database [Graveley et al., 2011; modENCODE Consortium et al., 2010]).

See also Figure S6.

- EXPERIMENTAL MODEL AND SUBJECT DETAILS
  - Drosophila Melanogaster Genetics
- METHOD DETAILS
  - Adult Haltere and Wing Preparations
  - Inmunohistochemistry
  - Ex Vivo Culture of Pupal Imaginal Wing Discs
  - Live-Imaging and Imaging of Fixed Samples
  - Rok Inhibitor Assay
  - Metalloprotease Treatment
  - Protease Inhibitor Treatment
  - Modeling of Convergent Extension. A Continuum Model for Autonomous Convergent Extension of the Imaginal Disc
- QUANTIFICATION AND STATISTICAL ANALYSIS
  - Quantification of Wing Size and Shape (Figures 1E, 3B, and S2C)
  - Quantification of Cell and Wing Elongation and Area (Figures 3D–3G, S2A, S2B, S2D–S2G, and S3)
  - Cell Segmentation
  - Quantification of Cell Rearrangements
  - Quantification ECM Degradation
  - Quantification of Myosin Intensity along the Apico-Basal Axis
  - Statistical Analysis

SUPPLEMENTAL INFORMATION

Supplemental Information includes seven figures and seven videos and can be found with this article online at https://doi.org/10.1016/j.devcel.2018.06.006.

ACKNOWLEDGMENTS

We thank A. Wordaz, G. Gibson, the Bloomington Drosophila Stock Center, the Drosophila Genomics Resource Center, and the Developmental Studies Hybridoma Bank for flies and/or reagents. This work was supported by The Francis Crick Institute, which receives its core funding from Cancer Research UK, the UK Medical Research Council, and the Wellcome Trust, which receives its core funding from Cancer Research UK (FC001180), the UK Medical Research Council (FC001180), and the Wellcome Trust (FC001180), as well as a Wellcome Trust Investigator award (102853/B/13/Z).

AUTHOR CONTRIBUTIONS

M.D.C.D.D.L.L designed and performed the experimental work and part of the data analysis (wing size and shape, cell rearrangements, and fluorescence quantification). R.P.R. contributed to the initial experimental work. P.S.G. segmented epithelial cells and performed the analysis of the segmented data and the continuum model. J.R.D., A.H., and N.T. developed the segmentation software. S.A. and G.S. supervised the segmentation analysis and the continuum model of wing elongation. B.J.T. designed the experiments and wrote the manuscript with input from the other authors.

DECLARATION OF INTERESTS

The authors declare no competing or financial interests.

Received: July 28, 2017
Revised: April 4, 2018
Accepted: June 7, 2018
Published: July 2, 2018

REFERENCES

Aldaz, S., Escudero, L.M., and Freeman, M. (2010). Live imaging of *Drosophila* imaginal disc development. Proc. Natl. Acad. Sci. USA *107*, 14217–14222.

Aldaz, S., Escudero, L.M., and Freeman, M. (2013). Dual role of myosin II during *Drosophila* imaginal disc metamorphosis. Nat. Commun. *4*, 1761.

Appel, L.F., Prout, M., Abu-Shumays, R., Hammonds, A., Garbe, J.C., Fristrom, D., and Fristrom, J. (1993). The *Drosophila* Stubble-stubbloid gene encodes an apparent transmembrane serine protease required for epithelial morphogenesis. Proc. Natl. Acad. Sci. USA *90*, 4937–4941.

Baena-Lopez, L.A., Baonza, A., and Garcia-Bellido, A. (2005). The orientation of cell divisions determines the shape of *Drosophila* organs. Curr. Biol. *15*, 1640–1644.

Beaton, A.H., Kiss, I., Fristrom, D., and Fristrom, J.W. (1988). Interaction of the stubble-stubbloid locus and the broad-complex of *Drosophila melanogaster*. Genetics *120*, 453–464.

Behrndt, M., Salbreux, G., Campinho, P., Hauschild, R., Oswald, F., Roensch, J., Grill, S.W., and Heisenberg, C.P. (2012). Forces driving epithelial spreading in zebrafish gastrulation. Science *338*, 257–260.

Bell, M.R., Thompson, L.M., Rodriguez, K., and Gore, A.C. (2016). Two-hit exposure to polychlorinated biphenyls at gestational and juvenile life stages: 1. Sexually dimorphic effects on social and anxiety-like behaviors. Horm. Behav. *78*, 168–177.

Bertet, C., Sulak, L., and Lecuit, T. (2004). Myosin-dependent junction remodelling controls planar cell intercalation and axis elongation. Nature *429*, 667–671.

Blankenship, J.T., Backovic, S.T., Sanny, J.S., Weitz, O., and Zallen, J.A. (2006). Multicellular rosette formation links planar cell polarity to tissue morphogenesis. Dev. Cell *11*, 459–470.

Bonnans, C., Chou, J., and Werb, Z. (2014). Remodelling the extracellular matrix in development and disease. Nat. Rev. Mol. Cell Biol. *15*, 786–801.

Butler, L.C., Blanchard, G.B., Kabla, A.J., Lawrence, N.J., Welchman, D.P., Mahadevan, L., Adams, R.J., and Sanson, B. (2009). Cell shape changes indicate a role for extrinsic tensile forces in *Drosophila* germ-band extension. Nat. Cell Biol. *11*, 859–864.

Calleja, M., Moreno, E., Pelaz, S., and Morata, G. (1996). Visualization of gene expression in living adult *Drosophila*. Science *274*, 252–255.

Campinho, P., Behrndt, M., Ranft, J., Risler, T., Minc, N., and Heisenberg, C.P. (2013). Tension-oriented cell divisions limit anisotropic tissue tension in epithelial spreading during zebrafish epiboly. Nat. Cell Biol. *15*, 1405–1414.

Chae, J., Kim, M.J., Goo, J.H., Collier, S., Gubb, D., Charlton, J., Adler, P.N., and Park, W.J. (1999). The *Drosophila* tissue polarity gene starry night encodes a member of the protocadherin family. Development *126*, 5421–5429.

Collinet, C., Rauzi, M., Lenne, P.F., and Lecuit, T. (2015). Local and tissue-scale forces drive oriented junction growth during tissue extension. Nat. Cell Biol. *17*, 1247–1258.

Concha, M.L., and Adams, R.J. (1998). Oriented cell divisions and cellular morphogenesis in the zebrafish gastrula and neurula: a time-lapse analysis. Development *125*, 983–994.

da Silva, S.M., and Vincent, J.P. (2007). Oriented cell divisions in the extending germband of *Drosophila*. Development *134*, 3049–3054.

Dreher, D., Pasakarnis, L., and Brunner, D. (2016). SnapShot: mechanical forces in development II. Cell *165*, 1028.e1.

Etournay, R., Popovic, M., Merkel, M., Nandi, A., Blasse, C., Aigouy, B., Brandl, H., Myers, G., Salbreux, G., Julicher, F., et al. (2015). Interplay of cell dynamics and epithelial tension during morphogenesis of the *Drosophila* pupal wing. Elife *4*, e07090.

Ettensohn, C.A. (1985). Gastrulation in the sea urchin embryo is accompanied by the rearrangement of invaginating epithelial cells. Dev. Biol. *112*, 383–390.

Fernandez-Gonzalez, R., Simoes Sde, M., Roper, J.C., Eaton, S., and Zallen, J.A. (2009). Myosin II dynamics are regulated by tension in intercalating cells. Dev. Cell *17*, 736–743.

Fernandez-Gonzalez, R., and Zallen, J.A. (2009). Cell mechanics and feedback regulation of actomyosin networks. Sci. Signal. *2*, pe78.

Fristrom, D. (1988). The cellular basis of epithelial morphogenesis. A review. Tissue Cell *20*, 645–690.

Fristrom, D., and Fristrom, J.W. (1975). The mechanism of evagination of imaginal discs of *Drosophila melanogaster*. 1. General considerations. Dev. Biol. *43*, 1–23.

Fritz, A.E., Ikmi, A., Seidel, C., Paulson, A., and Gibson, M.C. (2013). Mechanisms of tentacle morphogenesis in the sea anemone *Nematostella vectensis*. Development *140*, 2212–2223.

Gibson, W.T., Veldhuis, J.H., Rubinstein, B., Cartwright, H.N., Perrimon, N., Brodland, G.W., Nagpal, R., and Gibson, M.C. (2011). Control of the mitotic cleavage plane by local epithelial topology. Cell *144*, 427–438.

Godenschwege, T.A., Pohar, N., Buchner, S., and Buchner, E. (2000). Inflated wings, tissue autolysis and early death in tissue inhibitor of metalloproteinases mutants of *Drosophila*. Eur. J. Cell Biol. *79*, 495–501.

Gong, Y., Mo, C., and Fraser, S.E. (2004). Planar cell polarity signalling controls cell division orientation during zebrafish gastrulation. Nature *430*, 689–693.

Graveley, B.R., Brooks, A.N., Carlson, J.W., Duff, M.O., Landolin, J.M., Yang, L., Artieri, C.G., van Baren, M.J., Boley, N., Booth, B.W., et al. (2011). The developmental transcriptome of *Drosophila melanogaster*. Nature *471*, 473–479.

Guo, Y., Flegel, K., Kumar, J., McKay, D.J., and Buttitta, L.A. (2016). Ecdysone signaling induces two phases of cell cycle exit in *Drosophila* cells. Biol. Open *5*, 1648–1661.

Haigo, S.L., and Bilder, D. (2011). Global tissue revolutions in a morphogenetic movement controlling elongation. Science *331*, 1071–1074.

Hardin, J.D., and Cheng, L.Y. (1986). The mechanisms and mechanics of archenteron elongation during sea urchin gastrulation. Dev. Biol. *115*, 490–501.

Heisenberg, C.P., Tada, M., Rauch, G.J., Saude, L., Concha, M.L., Geisler, R., Stemple, D.L., Smith, J.C., and Wilson, S.W. (2000). Silberblick/Wnt11 mediates convergent extension movements during zebrafish gastrulation. Nature *405*, 76–81.

Huang, D.H., Su, L., Peng, X.H., Zhang, H., Khuri, F.R., Shin, D.M., and Chen, Z.G. (2009). Quantum dot-based quantification revealed differences in subcellular localization of EGFR and E-cadherin between EGFR-TKI sensitive and insensitive cancer cells. Nanotechnology *20*, 225102.

Irvine, K.D., and Wieschaus, E. (1994). Cell intercalation during *Drosophila* germband extension and its regulation by pair-rule segmentation genes. Development *120*, 827–841.

Karim, F.D., Guild, G.M., and Thummel, C.S. (1993). The *Drosophila* Broad-Complex plays a key role in controlling ecdysone-regulated gene expression at the onset of metamorphosis. Development *118*, 977–988.

Keller, R.E. (1980). The cellular basis of epiboly—an SEM study of deep-cell rearrangement during gastrulation in *Xenopus laevis*. J. Embryol. Exp. Morphol. *60*, 201–234.

Keller, R.E., and Trinkaus, J.P. (1987). Rearrangement of enveloping layer cells without disruption of the epithelial permeability barrier as a factor in *Fundulus* epiboly. Dev. Biol. *120*, 12–24.

Kiss, I., Beaton, A.H., Tardiff, J., Fristrom, D., and Fristrom, J.W. (1988). Interactions and developmental effects of mutations in the Broad-Complex of *Drosophila melanogaster*. Genetics *118*, 247–259.

Lan, H., Wang, Q., Fernandez-Gonzalez, R., and Feng, J.J. (2015). A biomechanical model for cell polarization and intercalation during *Drosophila* germband extension. Phys. Biol. *12*, 056011.

Lecuit, T., and Le Goff, L. (2007). Orchestrating size and shape during morphogenesis. Nature *450*, 189–192.

Lecuit, T., and Yap, A.S. (2015). E-cadherin junctions as active mechanical integrators in tissue dynamics. Nat. Cell Biol. *17*, 533–539.

Legoff, L., Rouault, H., and Lecuit, T. (2013). A global pattern of mechanical stress polarizes cell divisions and cell shape in the growing *Drosophila* wing disc. Development *140*, 4051–4059.

Lewis, E.B. (1978). A gene complex controlling segmentation in *Drosophila*. Nature *276*, 565–570.

Lienkamp, S.S., Liu, K., Karner, C.M., Carroll, T.J., Ronneberger, O., Wallingford, J.B., and Walz, G. (2012). Vertebrate kidney tubules elongate using a planar cell polarity-dependent, rosette-based mechanism of convergent extension. Nat. Genet. *44*, 1382–1387.

Lu, B., Usui, T., Uemura, T., Jan, L., and Jan, Y.N. (1999). Flamingo controls the planar polarity of sensory bristles and asymmetric division of sensory organ precursors in *Drosophila*. Curr. Biol. *9*, 1247–1250.

Lye, C.M., Blanchard, G.B., Naylor, H.W., Muresan, L., Huisken, J., Adams, R.J., and Sanson, B. (2015). Mechanical coupling between endoderm invagination and axis extension in *Drosophila*. Plos Biol. *13*, e1002292.

Lye, C.M., and Sanson, B. (2011). Tension and epithelial morphogenesis in *Drosophila* early embryos. Curr. Top. Dev. Biol. *95*, 145–187.

Mandaron, P. (1970). [In vitro development of imaginal disks of *Drosophila*. Morphological and histological aspects]. Dev. Biol. *22*, 298–320.

Mandaron, P. (1971). [Mechanism of imaginal disk evagination in *Drosophila*]. Dev. Biol. *25*, 581–605.

Mao, Y., Rauskolb, C., Cho, E., Hu, W.L., Hayter, H., Minihan, G., Katz, F.N., and Irvine, K.D. (2006). Dachs: an unconventional myosin that functions downstream of Fat to regulate growth, affinity and gene expression in *Drosophila*. Development *133*, 2539–2551.

Mao, Y., Tournier, A.L., Bates, P.A., Gale, J.E., Tapon, N., and Thompson, B.J. (2011). Planar polarization of the atypical myosin Dachs orients cell divisions in *Drosophila*. Genes Dev. *25*, 131–136.

Mao, Y., Tournier, A.L., Hoppe, A., Kester, L., Thompson, B.J., and Tapon, N. (2013). Differential proliferation rates generate patterns of mechanical tension that orient tissue growth. EMBO J. *32*, 2790–2803.

Matakatsu, H., and Blair, S.S. (2006). Separating the adhesive and signaling functions of the Fat and Dachsous protocadherins. Development *133*, 2315–2324.

Merkel, M., Etournay, R., Popovic, M., Salbreux, G., Eaton, S., and Julicher, F. (2017). Triangles bridge the scales: quantifying cellular contributions to tissue deformation. Phys. Rev. E *95*, 032401.

Milner, M.J., Bleasby, A.J., and Kelly, S.L. (1984). The role of the peripodial membrane of leg and wing imaginal discs of *Drosophila melanogaster* during evagination and differentiation in vitro. Wilhelm Roux Arch. Dev. Biol. *193*, 180–186.

modENCODE Consortium, Roy, S., Ernst, J., Kharchenko, P.V., Kheradpour, P., Negre, N., Eaton, M.L., Landolin, J.M., Bristow, C.A., Ma, L., et al. (2010). Identification of functional elements and regulatory circuits by *Drosophila* modENCODE. Science *330*, 1787–1797.

Paluch, E., and Heisenberg, C.P. (2009). Biology and physics of cell shape changes in development. Curr. Biol. *19*, R790–R799.

Pare, A.C., Vichas, A., Fincher, C.T., Mirman, Z., Farrell, D.L., Mainieri, A., and Zallen, J.A. (2014). A positional Toll receptor code directs convergent extension in *Drosophila*. Nature *515*, 523–527.

Pasakarnis, L., Dreher, D., and Brunner, D. (2016). SnapShot: mechanical forces in development I. Cell *165*, 754.e1.

Pastor-Pareja, J.C., and Xu, T. (2011). Shaping cells and organs in *Drosophila* by opposing roles of fat body-secreted Collagen IV and perlecan. Dev. Cell *21*, 245–256.

Pavlopoulos, A., and Akam, M. (2011). Hox gene Ultrabithorax regulates distinct sets of target genes at successive stages of *Drosophila* haltere morphogenesis. Proc. Natl. Acad. Sci. USA *108*, 2855–2860.

Poodry, C.A., and Schneiderman, H.A. (1970). The ultrastructure of the developing leg of *Drosophila melanogaster*. Wilhelm Roux Arch. Dev. Biol. *166*, 1–44.

Popovic, M., Nandi, A., Merkel, M., Etournay, R., Eaton, S., Julicher, F., and Salbreux, G. (2017). Active dynamics of tissue shear flow. New J. Phys. *19*, https://doi.org/10.1088/1367-2630/aa5756.

Rauzi, M., Verant, P., Lecuit, T., and Lenne, P.F. (2008). Nature and anisotropy of cortical forces orienting *Drosophila* tissue morphogenesis. Nat. Cell Biol. *10*, 1401–1410.

Ray, R.P., Matamoro-Vidal, A., Ribeiro, P.S., Tapon, N., Houle, D., Salazar-Ciudad, I., and Thompson, B.J. (2015). Patterned anchorage to the apical

extracellular matrix defines tissue shape in the developing appendages of *Drosophila*. Dev. Cell *34*, 310–322.

Riddiford, L.M. (1993). Hormone receptors and the regulation of insect metamorphosis. Receptor *3*, 203–209.

Rodriguez-Fraticelli, A.E., and Martin-Belmonte, F. (2014). Picking up the threads: extracellular matrix signals in epithelial morphogenesis. Curr. Opin. Cell Biol. *30*, 83–90.

Royou, A., Sullivan, W., and Karess, R. (2002). Cortical recruitment of nonmuscle myosin II in early syncytial *Drosophila* embryos: its role in nuclear axial expansion and its regulation by Cdc2 activity. J. Cell Biol. *158*, 127–137.

Saburi, S., Hester, I., Fischer, E., Pontoglio, M., Eremina, V., Gessler, M., Quaggin, S.E., Harrison, R., Mount, R., and McNeill, H. (2008). Loss of Fat4 disrupts PCP signaling and oriented cell division and leads to cystic kidney disease. Nat. Genet. *40*, 1010–1015.

Sarov, M., Barz, C., Jambor, H., Hein, M.Y., Schmied, C., Suchold, D., Stender, B., Janosch, S., K J, VV., et al. (2016). A genome-wide resource for the analysis of protein localisation in *Drosophila*. Elife *5*, e12068.

Saxena, A., Denholm, B., Bunt, S., Bischoff, M., VijayRaghavan, K., and Skaer, H. (2014). Epidermal growth factor signalling controls myosin II planar polarity to orchestrate convergent extension movements during *Drosophila* tubulogenesis. PLoS Biol. *12*, e1002013.

Schindelin, J., Arganda-Carreras, I., Frise, E., Kaynig, V., Longair, M., Pietzsch, T., Preibisch, S., Rueden, C., Saalfeld, S., Schmid, B., et al. (2012). Fiji: an open-source platform for biological-image analysis. Nat. Methods *9*, 676–682.

Sepich, D.S., Calmelet, C., Kiskowski, M., and Solnica-Krezel, L. (2005). Initiation of convergence and extension movements of lateral mesoderm during zebrafish gastrulation. Dev. Dyn. *234*, 279–292.

Simoes Sde, M., Blankenship, J.T., Weitz, O., Farrell, D.L., Tamada, M., Fernandez-Gonzalez, R., and Zallen, J.A. (2010). Rho-kinase directs Bazooka/Par-3 planar polarity during *Drosophila* axis elongation. Dev. Cell *19*, 377–388.

Solnica-Krezel, L. (2005). Conserved patterns of cell movements during vertebrate gastrulation. Curr. Biol. *15*, R213–R228.

Solon, J., Kaya-Copur, A., Colombelli, J., and Brunner, D. (2009). Pulsed forces timed by a ratchet-like mechanism drive directed tissue movement during dorsal closure. Cell *137*, 1331–1342.

Taylor, J., Abramova, N., Charlton, J., and Adler, P.N. (1998). Van Gogh: a new *Drosophila* tissue polarity gene. Genetics *150*, 199–210.

Vichas, A., and Zallen, J.A. (2011). Translating cell polarity into tissue elongation. Semin. Cell Dev. Biol. *22*, 858–864.

Voiculescu, O., Bertocchini, F., Wolpert, L., Keller, R.E., and Stern, C.D. (2007). The amniote primitive streak is defined by epithelial cell intercalation before gastrulation. Nature *449*, 1049–1052.

Wang, Y.C., Khan, Z., Kaschube, M., and Wieschaus, E.F. (2012). Differential positioning of adherens junctions is associated with initiation of epithelial folding. Nature *484*, 390–393.

Ward, R.E., Evans, J., and Thummel, C.S. (2003). Genetic modifier screens in *Drosophila* demonstrate a role for Rho1 signaling in ecdysone-triggered imaginal disc morphogenesis. Genetics *165*, 1397–1415.

Wei, Y., and Mikawa, T. (2000). Formation of the avian primitive streak from spatially restricted blastoderm: evidence for polarized cell division in the elongating streak. Development *127*, 87–96.

Wilkin, M.B., Becker, M.N., Mulvey, D., Phan, I., Chao, A., Cooper, K., Chung, H.J., Campbell, I.D., Baron, M., and MacIntyre, R. (2000). *Drosophila* dumpy is a gigantic extracellular protein required to maintain tension at epidermal-cuticle attachment sites. Curr. Biol. *10*, 559–567.

Wolff, T., and Rubin, G.M. (1998). Strabismus, a novel gene that regulates tissue polarity and cell fate decisions in *Drosophila*. Development *125*, 1149–1159.

Xiong, F., Ma, W., Hiscock, T.W., Mosaliganti, K.R., Tentner, A.R., Brakke, K.A., Rannou, N., Gelas, A., Souhait, L., Swinburne, I.A., et al. (2014). Interplay of cell shape and division orientation promotes robust morphogenesis of developing epithelia. Cell *159*, 415–427.

Zallen, J.A., and Wieschaus, E. (2004). Patterned gene expression directs bipolar planar polarity in *Drosophila*. Dev. Cell *6*, 343–355.

STAR★METHODS

KEY RESOURCES TABLE

REAGENT or RESOURE	SOURCE	IDENTIFIER
Antibodies		
Goat polyclonal anti-GFP	Abcam	Cat# ab6662; RRID: AB_305635
Rabbit anti-Bazooka	Andreas Wordaz laboratory	RRID: AB_2570125
Mouse anti-Armadillo	DSHB	Cat# 25E9.D7; RRID: AB_528104
Mouse anti-MMP1	DSHB	Cat# 3A6B4; RRID: AB_579780
Mouse anti-MMP1	DSHB	Cat# 3B8D12; RRID: AB_579781
Mouse anti-MMP1	DSHB	Cat# 5H7B11; RRID: AB_579779
Mouse anti-Ubx	DSHB	Cat# FP3.38; RRID: AB_10805300
Goat anti-mouse Alexa fluor 546	Invitrogen	Cat# A11030; RRID: AB_144695
Goat anti-rabbit Alexa fluor 546	Invitrogen	Cat# A11035; RRID: AB_143051
Chemicals, Peptides, and Recombinant Proteins		
Shield and Sang M3 insect medium	Sigma	S365
2% Fetal bovine serum	Sigma	F3018
Streptomycin/ampicillin antibiotics mix	Invitrogen	15140-122
Ecdysone	Sigma	E9004
Insulin	Sigma	5500
Methyl-cellulose	Sigma	M0387
Rock inhibitor	Sigma	Y-27632
Trypsin-EDTA	Gibco	15400-054
DAPI	Sigma	D9542
Phalloidin-Atto 647N	Sigma	65906
Experimental Models: Organisms/Strains		
D. melanogaster: E-cadherin-GFP.<br>*w; shg-GFP*	Huang et al., 2009	N/A
D. melanogaster: Dumpy-YFP.<br>$w^{[1118]}$; *PBac{681.P.FSVS-1}dp[CPTI001769]*	DGGR (Kyoto)	Cat# 115238; RRID: DGGR 115238
D. melanogaster: Vkg-GFP.<br>$w^{[*]}$; *P{PTT-un1}*vkg^{G205}	FlyTrap	G205
D. melanogaster: Lanβ1-GFP.<br>$w^{[*]}$;; *LanB1-GFP/TM2*	Sarov et al., 2016	N/A
D. melanogaster: Perlecan-GFP.<br>$w^{[*]}$ *P{*w^{+mC}*=PTT-un1}ZCL1700*	DGGR (Kyoto)	Cat# 110807; RRID: DGGR_110807
D. melanogaster: Myosin-II-GFP.<br>*sqhAX3; sqh-Sqh-GFP*	Royou et al., 2002	N/A
D. melanogaster: MMP2 overexpression.<br>$w^{[*]}$; *P{w[+mC]=UAS-Mmp2.P}2*	Bloomington	Cat# 58705; RRID: BDSC_58705
D. melanogaster: *Timp* overexpression.<br>$w^{[*]}$; *P{w[+mC]=UAS-Timp.P}3*	Bloomington	Cat# 58708; RRID: BDSC_58708
D. melanogaster: Ubx^{la} allele overexpression.<br>$w^{[*]}$; *P{w[+mC]=UAS-*$Ubx^{la.C}$*}36.2/TM3, Ser[1]*	Bloomington	Cat# 911; RRID: BDSC_911
D. melanogaster: RNAi of Bazooka.<br>$w^{[*]}$;; $baz^{NIG.5055R}$ */TM3*	NIG-Fly	5055R-1
D. melanogaster: RNAi of Ultrabithorax<br>$w^{[1118]}$; *P{GD5049}v37823*	VDRC	Cat# 37823; RRID: Flybase_FBst0462184
D. melanogaster: Rok^{K116A} allele overexpression<br>$w^{[*]}$; *UAS.venus-*Rok^{K116A} */TM6B*	Simoes Sde et al., 2010	N/A
D. melanogaster: Dll-Gal4<br>$w^{[*]}$; *Dll-Gal4/CyO*	Calleja et al., 1996	N/A

(*Continued on next page*)

Continued		
REAGENT or RESOURE	SOURCE	IDENTIFIER
D. melanogaster: nubbin-Gal4<br>*w[*]; nubbin-GAL4*	Calleja et al., 1996	N/A
D. melanogaster: Ubx-Gal4<br>*w[*];; Ubx-Gal4/TM6B*	GregoryGibson	N/A
D. melanogaster: Fat^{8} mutant allele<br>*w[*]; FRT40A* ft^{GrV}*/CyOGFP; AFZ/TM2*	Mao et al., 2006	N/A
D. melanogaster: Fat^{GrV} mutant allele<br>*w[*]; FRT40A* ft^{-GrV} */CyOGFP; AFZ/TM2*	Matakatsu and Blair, 2006	N/A
Software and Algorithms		
Fiji	Schindelin et al., 2012	RRID: SCR 002285

CONTACT FOR REAGENT AND RESOURCE SHARING

Further information and requests for resources and reagents should be directed to and will be fulfilled by the Lead Contact, Barry J. Thompson (barry.thompson@crick.ac.uk).

EXPERIMENTAL MODEL AND SUBJECT DETAILS

Drosophila Melanogaster Genetics

Flies were grown at 25 C using standard procedures. The following fluorescent-tagged proteins were used: E-cadherin-GFP (Huang et al., 2009), Dp-YFP (Drosophila Genomics and Genetic Resources (Kyoto), 115238), Collagen IV-GFP (α2-subunit, Vkg-GFP; FlyTrap G205), Laminin-GFP (Laminin β1-subunit, Lanβ1-GFP; (Sarov et al., 2016)) Perlecan-GFP (Pcan-GFP, Kyoto DGGR, 110807) and Myosin-II-GFP (*sqh-GFP* construct) in the sqh^{AX3} null mutant background (Royou et al., 2002). Gene expression mediated by the *Gal4/UAS* system was performed at 25 C. To decrease *Ubx* levels in the haltere, *UAS.UbxRNAi* (VDRC, 37823) was combined with the *Ubx-Gal4* driver (kindly provided by Gregory Gibson). To ectopically express *Ubx* or *bazooka* in the wing, the *UAS-*Ubx^{la} (Bloomington, 911, (Pavlopoulos and Akam, 2011)) or *UAS.bazRNAi* constructs (NIG-Fly, 5055R-1) were combined with the *nubbin-Gal4 (nub-GAL4*) driver, respectively. Overexpression of *MMP2* in the haltere was mediated by combining the *UAS.MMP2* construct (Bloomington, 58705) and *Ubx-GAL4*; and *Timp* was overexpressed in the wing by combining *UAS.Timp* (Bloomington, 58708) and *nub-GAL4*. *UAS.venus-*Rok^{K116A} (Simoes Sde et al., 2010) expression was mediated using specific drivers for the different imaginal discs: *nub-Gal4* in the wing, *Dll-Gal4* (Calleja et al., 1996) in the leg, and *Ubx-Gal4* in the haltere. Fat mutant condition consists of the heterozygous combination of the loss of function alleles Fat^{8} and Fat^{GrV} (Mao et al., 2006; Matakatsu and Blair, 2006).

METHOD DETAILS

Adult Haltere and Wing Preparations

Halteres and wings were dissected from the adult fly, fixed in 3:1 ethanol glycerol and mounted in Hoyer's mounting media. Images were acquired on a Zeiss axioplan microscope using a LeicaDFC420c digital camera and processed with Adobe Photoshop software.

Inmunohistochemistry

White pupae were collected and aged, and then imaginal discs were dissected from the puparium in PBS and transferred to 4% paraformaldehyde for fixation. After 30 minutes of fixation, tissues were immunostained as described in (Ray et al., 2015). Anti-GFP antibody (Abcam, ab6662, 1:400) was used to amplify E-cad-GFP, Dp-YFP, Vkg-GFP, Lanβ1-GFP, Pcan-GFP, and Myosin-II-GFP immunofluorescence signals; rabbit anti-Bazooka was used at 1:250 (A. Wordaz), mouse anti-Armadillo at 1:100 (DSHB), mouse anti-Broad at 1:100 (DSHB, 25E9.D7), mouse anti-MMP1 1:1:1 antibodies cocktail (DSHB; 3A6B4, 3B8D12 and 5H7B11) at a 1:4, and mouse anti-Ubx at 1:10 (DSHB, FP3.38). Secondary antibodies, goat Alexa fluor 488, 546 or 647 (Invitrogen), were used at 1:500. DAPI and rhodamine phallodin (Sigma, 65906) were used at 1:250. Samples were mounted in Vectashield (Vector Labs, H1000) using different separators depending on the thickness of the sample.

Ex Vivo Culture of Pupal Imaginal Wing Discs

Pupal wing discs of the appropriate age were cultured as described in (Bell et al., 2016; Aldaz et al., 2010). Wing discs were dissected from the puparium in Shield and Sang M3 insect medium (Sigma, S3652) supplemented with 2% fetal bovine serum (Sigma, F3018), 1% streptomycin/ampicillin antibiotics mix (Invitrogen, 15140-122), 0.1 μg/mL ecdysone (Sigma, E9004) and 0.14 μg/mL insulin

(Sigma, I5500). For imaging, wing discs were transferred to a tissue culture dish (Fluorodish, FD35) containing supplemented Shield and Sang medium plus 2.5% methyl-cellulose (Sigma, M0387).

Live-Imaging and Imaging of Fixed Samples

In vivo and *ex vivo* samples images were acquired with a Leica SP5 confocal using 20x/ 0.70 NA or 40x/ 1.25 NA immersion objectives, controlled by the Leica Las AF software. Alternatively, a Zeiss LSM 880 confocal controlled by the ZEN software was used to perform high-resolution live imaging experiments, using a 40x/ 1.3 NA and applying a 2x zoom magnification. Images were analysed and processed using Fiji, Adobe Photoshop and Adobe Illustrator software. Live imaging experiments were performed at room temperature and an average of 50 Z-sections at 1 to 2 μm interval were acquired every 5 minutes.

Rok Inhibitor Assay

To inhibit myosin contraction during convergent extension, 4 hours APF sqh^{AX3}; {*sqh-GFP*} wing discs were dissected and transferred, right after peripodial membrane release, to supplemented Shield and Sang medium plus 2.5% methyl-cellulose containing 2.5mM Rock inhibitor (Sigma, Y-27632), and filmed immediately, or alternatively fixed and immunostained after 30 min of inhibitor treatment at 25 C. To inhibit myosin contraction during wing expansion, 5 hours 30 min *sqhAX3*; {*sqh-GFP*} wing discs were dissected and transferred to supplemented Shield and Sang medium containing 2.5mM Rock inhibitor, incubated for 3 hours at 25C, fixed and immunostained.

Metalloprotease Treatment

Dp-YFP and Vkg-GFP haltere imaginal discs of the correct age were dissected from the puparium in PBS and transferred to PBS containing 0.001% Trypsin-EDTA (Gibco, 15400-054). Tissues were treated for 15 minutes at room temperature, and transferred to supplemented Shield and Sang medium to stop the trypsin reaction, fixed and immunostained.

Protease Inhibitor Treatment

dp-YFP,*nub-Gal4>Timp* and *vkg-YFP*,*nub-Gal4>Timp* wing imaginal discs were dissected from the puparium at 4 hours APF and transferred to supplemented Shield and Sang medium including a protease inhibitors mixture (2ml of media containing 1 tablet of cOmplete Protease Inhibitor Cocktail, 04693116001, Roche). Wing disc were incubated for 3 hours and 30 mminutes at 25 C, fixed and immunostained.

Modeling of Convergent Extension. A Continuum Model for Autonomous Convergent Extension of the Imaginal Disc

We discuss here a simple physical description of the pupal wing elongation occurring in between 4 and 7h APF. We use the framework developed in (Popovic et al., 2017) to compute the predicted time evolution of cell and tissue anisotropy.

1. Physical Description of Wing Elongation

For simplicity, the wing is described as a rectangular piece of tissue with length l and height h, subjected to spatially uniform shear (Figure S3C). We denote by x the proximal-distal axis and y the anterior-posterior axis. The deformation of the pupal wing is caused by shape changes of the cells as well as cellular rearrangements. We define $\tilde{v}_{ij}$ the traceless part of the gradient of flow of the wing and $\langle Q_{ij}\rangle$ the average cell elongation in the wing. We assume here that the shear occurs entirely along the proximal-distal and anterior-posterior axis, such that non-diagonal elements of $\tilde{v}_{ij}$ vanish. The traceless part of the gradient of flow describes the rate of tissue anisotropic deformation and can be decomposed into a contribution from cell elongation change and a contribution from cellular rearrangements (Popovic et al., 2017):

$$\tilde{v}_{ij} = \frac{d\langle Q_{ij}\rangle}{dt} + R_{ij} \qquad \text{(Equation 1)}$$

with R_{ij} a tensor of shear due to cellular rearrangements. We assume here that cellular rearrangements are entirely driven by myosin cell anisotropy, such that

$$R_{ij} = \lambda q_{ij}. \qquad \text{(Equation 2)}$$

with q_{ij} the nematic tensor characterizing myosin cellular anisotropy, and λ is a rate characterizing the response of shear due to cellular rearrangement to myosin polarization. We write the following equation for the constitutive equation for the traceless part of the stress, $\tilde{\sigma}_{ij} = \sigma_{ij} - \frac{1}{2}\sigma_{kk}\delta_{ij}$:

$$\tilde{\sigma}_{ij} = 2K\langle Q_{ij}\rangle + \zeta q_{ij} + 2\mu\frac{d\langle Q_{ij}\rangle}{dt}. \qquad \text{(Equation 3)}$$

In Equation 3, the coefficient K is a cellular elastic modulus and the coefficient ζ characterizes active stresses arising from myosin anisotropic distribution in the cell. The last term is a viscous contribution with coefficient of viscosity μ which ensures that under free boundary conditions, the cell shape relaxes in finite time with a timescale $\tau_s = \mu/K$.

The coefficients K, ζ, μ , and λ are assumed to be constant, while the myosin anisotropic tensor q_{ij} varies with time. To describe the expansion of the pupal wing, we assume that the tissue is free to expand, such that $\tilde{\sigma}_{ij} = 0$ Therefore Equation 3 reduces to

$$2K\langle Q_{ij}\rangle + \zeta q_{ij} + 2\mu \frac{d\langle Q_{ij}\rangle}{dt} = 0, \qquad \text{(Equation 4)}$$

which can be solved for $\langle Q_{xx}\rangle(t)$:

$$\langle Q_{xx}\rangle(t) = e^{-\frac{t}{\tau_s}} \left(\langle Q_{xx}\rangle(t_0) e^{\frac{t_0}{\tau_s}} - \frac{\zeta}{2K} \int_{t_0}^{t} dt' e^{t'/\tau_s} q_{xx}(t') \right). \qquad \text{(Equation 5)}$$

The anisotropic diagonal component of the velocity gradient $\tilde{v}_{xx}$ is related to the natural strain rate in the tissue:

$$\tilde{v}_{xx} = \frac{1}{2}\partial_t (L - H) \qquad \text{(Equation 6)}$$

with

$$L = \log\left(\frac{l}{l_0}\right), \quad H = \log\left(\frac{h}{h_0}\right) \qquad \text{(Equation 7)}$$

where ***l*** and ***h*** are the length and height of the tissue respectively and $\boldsymbol{l}_0$ and $\boldsymbol{h}_0$ the corresponding values at $t = t_0$.

Using Equations 1 and 4, the natural strain rate of the tissue is then given by:

$$(L - H)(t) = (L - H)(t_0) - 2\int_{t_0}^{t} dt' \left(\frac{1}{\tau_s} \langle Q_{xx}\rangle(t') + \left(\frac{\zeta}{2\mu} - \lambda \right) q_{xx}(t') \right). \qquad \text{(Equation 8)}$$

2. Adjustment to Experimental Data

We choose the following form for the evolution of the myosin anisotropy $q_{xx}(t)$:

$$q_{xx}(t) = 1 \quad t_0 < t < t_1 \qquad \text{(Equation 9)}$$

$$q_{xx}(t) = \frac{t_2 - t}{t_2 - t_1} \quad t_1 < t < t_2 \qquad \text{(Equation 10)}$$

$$q_{xx}(t) = 0 \quad t > t_2 \qquad \text{(Equation 11)}$$

with t_0 = 4h APF, t_1 = 5h h APF and t_2 = 6h APF. This choice of time evolution of the magnitude of q_{xx} is in accordance with measurements of Myosin-II anisotropy (Figure S3A).

We then varied the parameter τ_s within a range of values that are physiologically relevant (from 0.1 to 1 hour). For each value of τ_s, we calculated the average cell elongation using Equations 5 and 8, taking the initial values $\langle Q_{xx}\rangle(t_0)$ to be equal to the experimental measured average cell elongation at t = 4h APF, and $(L-H)(t_0) = 0$. We then fitted the parameters $\zeta/2K$ and λ to experimental measurements of cell and tissue elongation (Figure S3D).

Figure S3E shows the parameter values obtained for $\zeta/2K$ and λ from the fits as a function of τ_s. We find that the parameter λ takes a value around $\sim 0.1\text{h}^{-1}$ for all values of τ_s. The value of $\zeta/2K$ is negative and varies between -0.06 and -0.14. The negative sign of the coefficient ζ corresponds to an overall anisotropic stress driving a contractile force along the anterior-posterior direction. This sign is consistent with experimentally observed myosin polarization along junctions oriented along the anterior-posterior direction.

QUANTIFICATION AND STATISTICAL ANALYSIS

Quantification of Wing Size and Shape (Figures 1E, 3B, and S2C)

Up to 8 wing discs of each stage were analysed, and DNA and actin cytoskeleton dyes (DAPI and rhodamine phalloidin) allowed us to detect wing shape. The entire wing disc was imaged by acquiring Z stacks each 1.72 microns. After generating the maximum intensity projection from the Z stacks, wing area, maximal length l (along PD axis), and width h (along AP axis), were calculated manually using the ROI tool from Fiji. The average height of each wing was calculated manually using measurements from different regions of the epithelium in apico-basal cross sections, using the ROI tool from Fiji.

Quantification of Cell and Wing Elongation and Area (Figures 3D–3G, S2A, S2B, S2D–S2G, and S3)

Cell area was quantified from cell segmentation of fixed samples by averaging the area of all segmented cells. Wing area was measured in Fiji taking the whole blade as the region of interest. Cell elongation was obtained by triangulating the network of cell junctions, as described in (Merkel et al., 2017; Etournay et al., 2015). Briefly, triangles were obtained by connecting the cell centers of neighbouring cells. Triangular shapes were compared to the shape of an equilateral triangle, resulting in the definition of a tensor of nematic elongation $\boldsymbol{Q}$. The tensor of nematic elongation was then averaged over all triangles to obtain the average elongation $\langle Q\rangle$

(Figure S2E). Alternatively, to obtain a measure of overall cell shape anisotropy, the magnitude of tensor $|\boldsymbol{Q}| = \sqrt{(Q_{xx})^2 + (Q_{xy})^2}$ was averaged over all triangles to obtain an average cell shape anisotropy $|\langle \boldsymbol{Q} \rangle|$ (Figure S3G).

Tissue elongation was obtained by defining the natural strain variables $L = \log(l/l_0)$ and $H = \log(h/h_0)$, with l_0 and h_0 the length and width at 4 hours APF, and taking the difference $L-H$ (Figure S2D).

The difference between tissue elongation and average cell elongation defines a cell rearrangement tensor $\boldsymbol{R}$, whose diagonal component along the x (proximal-distal) axis is given by:

$$R_{xx} = \frac{1}{2}\frac{d(L-H)}{dt} - \frac{d\langle Q_{xx} \rangle}{dt}$$

which corresponds to the contribution of cellular rearrangements to the deformation of the tissue along the proximal-distal axis. The data was obtained from 2, 3, 6 and 2 fixed wings disc samples at 4, 5, 6 and 7 hours APF, respectively.

In live imaging experiments, wing and cell area (n = 10) were calculated manually using the freehand ROI tool from Fiji.

Cell Segmentation

Segmentation was performed in Wolfram Mathematica, whereby images were initially blurred through convolution with a Shen-Castan matrix, in order to smooth pixel intensities whilst preserving edges. Cell membranes were highlighted with a ridge filter, which is a principal-curvature based region detector, and the cells segmented with a watershed algorithm. For triangulation analysis cells were highlighted with a unique hexadecimal colour code that was randomly generated. After the segmentation step, an interactive skeleton correction process was introduced. For this, a new graphical tool was developed in MATLAB (Mathworks Inc.) which allowed overlaying the original and the segmented image sequences with the purpose of making corrections by drawing or deleting skeleton segments in an easy and intuitive manner. The editing options were accessible through keyboard shortcuts. This allowed for efficient switching between editing options while advancing through the sequence. The actual drawing or deletion of skeleton segments could be performed with a Wacom Pen and Graphics Tablet which made this process highly intuitive. The corrected skeletons were then exported for the subsequent analysis.

Quantification of Cell Rearrangements

Four high resolution videos of E-cadherin-GFP wing discs cultured *ex vivo* from 4 hours 30 min to 5 hours APF (convergent extension), and from 6 hours 30 min to 7 hours APF (expansion) were analysed. The number of cells involved in cell rearrangements, as well as the type of event (T1, rosettes and T2) and the total number of cells (>1500 cells per wing) were manually analysed using Image J.

Quantification ECM Degradation

To analyse ECM degradation in the different tissues and experimental conditions, up to 12 imaginal discs were analysed. Several confocal Z stacks acquired each 1.72 microns were selected and projected to include the complete epithelial surface and the adjacent ECM, and their areas were calculated manually using ROI measurement tool in Fiji. The percentage of apical or basal epithelial surfaces (marked by actin), covered by apical Dp-YFP or basal Vkg-GFP, Lanβ1-GFP and Pcan-GFP ECM components were calculated.

Quantification of Myosin Intensity along the Apico-Basal Axis

To analyse how Myosin-II distributes along the epithelial apico-basal axis in the imaginal wing disc, apico-basal cross sections of the appropriate stages were acquired by confocal scanning in ZXY, and up to 20 cells were analysed. *sqh-GFP* fluorescence intensity was measured along linear ROIs perpendicular to the apico-basal axis, and separated approximately each 2 μm, using Fiji. Intensity values along each ROI were normalised in respect with the average of total intensity fluorescence to calculate the average fluorescence for each ROI. To analyse the planar polarisation of myosin along the AP axis in the wing disc, apical cross sections were acquired from 5 to 8 hours APF, and up to 20 cells were analysed. *sqh-GFP* fluorescence intensity at the edges of each cell was calculated manually using Fiji, and the fluorescence intensity corresponding to the boundaries along the AP axis was normalised in respect with the ones parallel to the PD axis.

Statistical Analysis

Experiments were performed with at least three biological replicas. The average, standard deviation and individual data points were represented. To determine which values were significantly different, p values were obtained using two-tailed Student's t tests. $p<0.05$, statistically significant; $p<0.005$, very statistically significant; $p<0.00$, extremely statistically significant.

Developmental Cell, Volume *46*

Supplemental Information

Apical and Basal Matrix Remodeling Control Epithelial Morphogenesis

Maria-del-Carmen Diaz-de-la-Loza, Robert P. Ray, Poulami S. Ganguly, Silvanus Alt, John R. Davis, Andreas Hoppe, Nic Tapon, Guillaume Salbreux, and Barry J. Thompson

<u>Supplemental material:</u>

Supplemental Figures S1-S7

Figure S1. Broad, MMP1 and Ultrabithorax localisation from late larvae to early pupal stages.

Figure S2. Analysis of cell anisotropy, cell rearrangements and cell area during convergent extension and expansion.

Figure S3. Quantification of Myosin-II localisation and a continuum model of convergent extension in the pupal wing.

Figure S4. The Fat cadherin system and the polarity protein Bazooka are not essential for anisotropic extension of the pupal wing.

Figure S5. Perlecan localisation in 7 hours APF legs.

Figure S6. Myosin-II localisation in the leg and the haltere.

Figure S7. Cellular attachment to the basal ECM and concomitant detachment from the apical ECM leads to the folding of the epithelia.

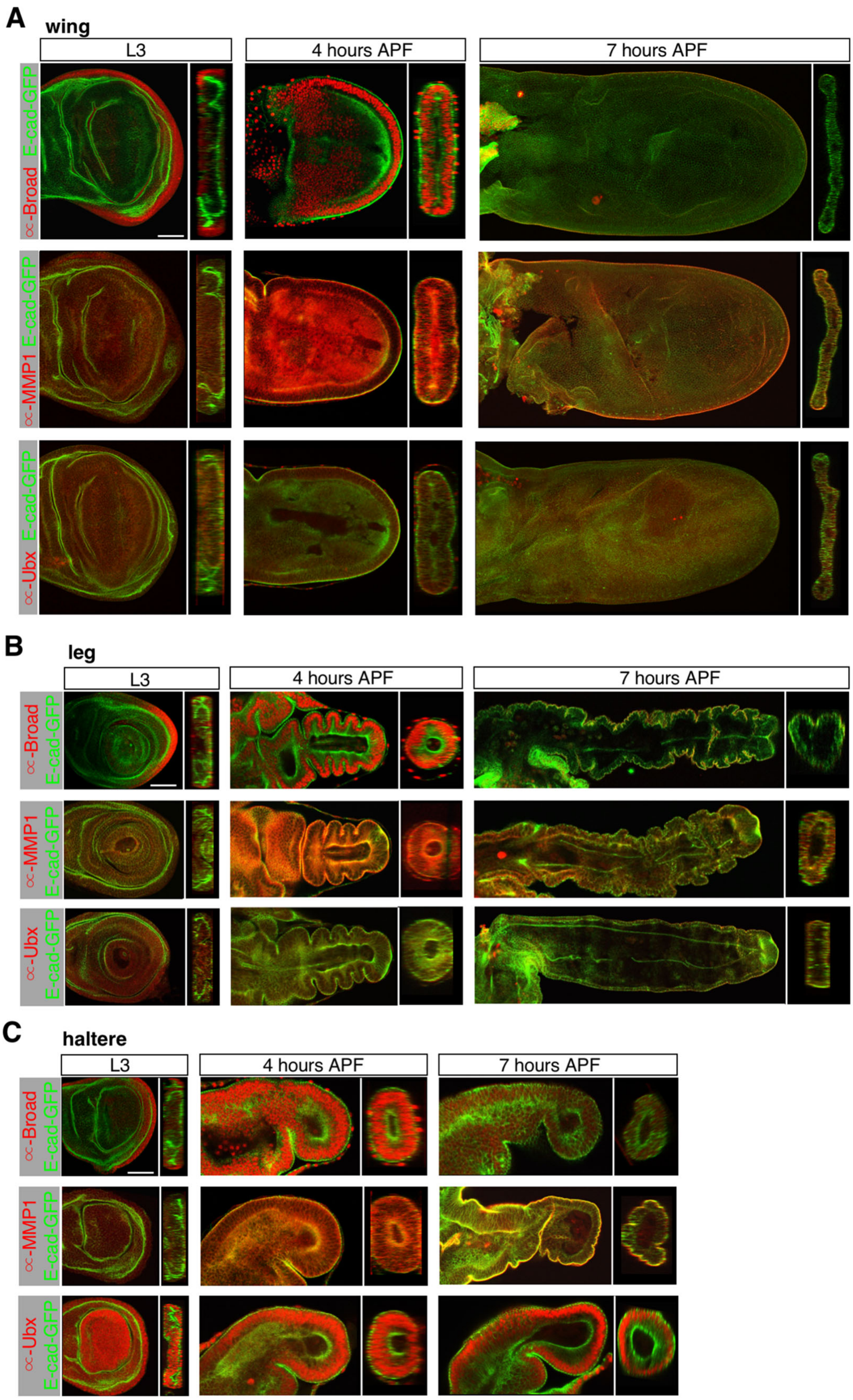

Figure S1. Broad, MMP1 and Ultrabithorax localisation from late larvae to early pupal stages. Related to Figure 2. Cross sections of developing wings (A), legs (B) and halteres (C), at third instar larvae stages (L3), 4 and 7 hours APF expressing *E-cadherin-GFP* (E-cad-GFP). Expression of *broad*, *MMP1* metalloprotease, and *Ultrabithorax* (*Ubx*) was detected by immunostaining. *broad* and *MMP1* are strongly expressed in all imaginal disc epithelia at 4 hours APF, but are almost no detectable at 7 hours APF. *Ubx* is exclusively expressed in the haltere epithelia. Although basal extracellular matrix (ECM) is not degraded in the haltere, MMP1 can be detected at 4 hours APF, suggesting that MMP1 activity should be inhibited in that tissue, most likely by the tissue inhibitor of metalloproteases (Timp). E-cad-GFP is shown in green and Broad, MMP1 and Ubx are shown in red. Scale bar, 50 µm.

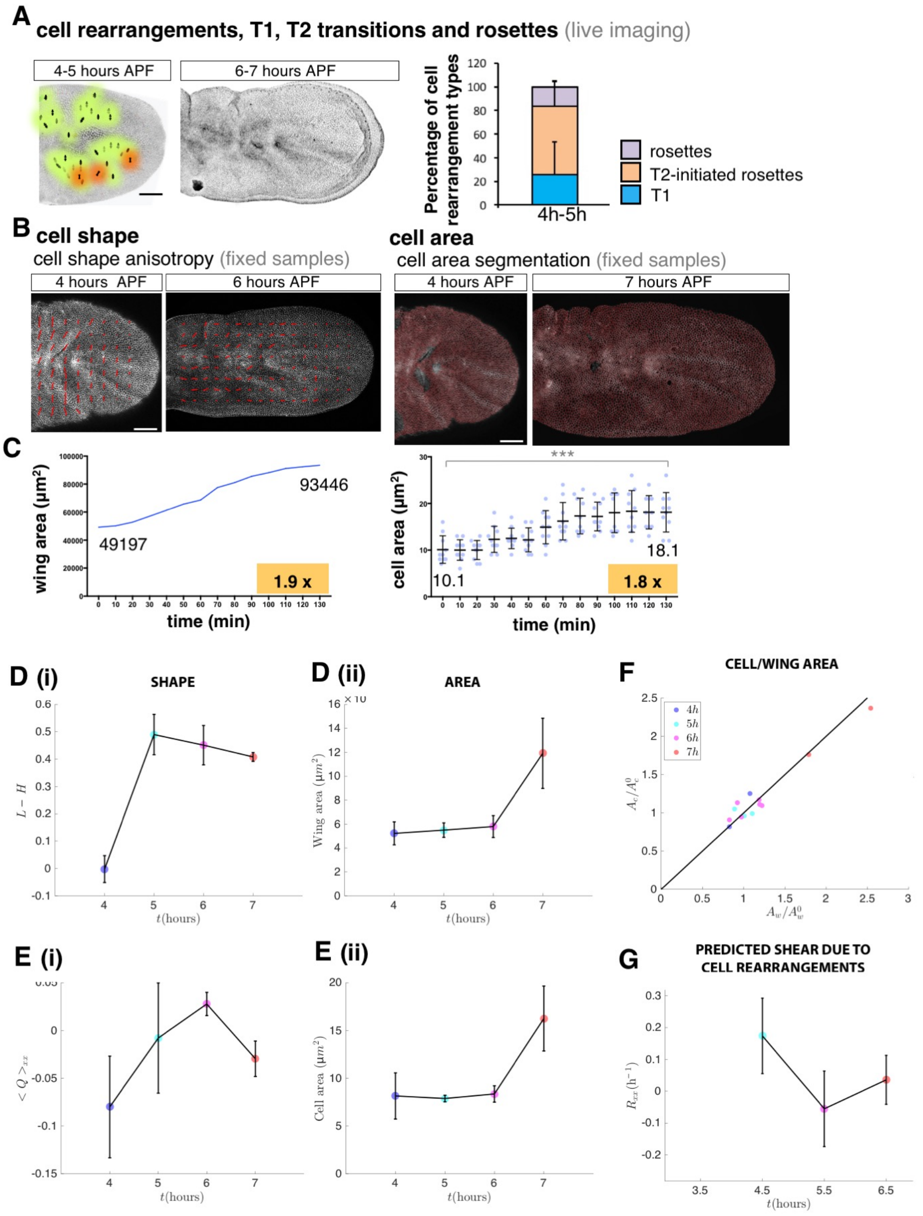

Figure S2. Analysis of cell anisotropy, cell rearrangements and cell area during convergent extension and expansion. Related to Figure 3.

(A-B) Cell rearrangements and cell shape and cell size changes were detected by live imaging experiments in *E-cad-GFP* expressing wings. (A) Cell intercalations were only present during convergent extension, mostly consisting in the shrinkage of junctions in the same direction of wing contraction through T1, T2 and rosettes that were often initiated after a T2 transition event (right, green shadowed arrows; red arrows indicate intercalation in the opposite direction). Average and standard deviation are represented for each type of cell rearrangement, n=4 wings, >1500 cells per wing. (B) Cell size and shape were analysed through segmentation on E-cad-GFP fixed samples from 4 to 7 hours APF. Red lines indicate the intensity and the direction of cell anisotropy (left) and the cell apical perimeter (right). Scale bar, 50 µm (left).

(C) Quantification of cell area increase during wing expansion in live imaging experiments, from 6 to 8 hours APF (Movie S1). Average and standard deviation of apical cell area (n=10) are represented, and statically significant differences are indicated (***p<0.001).

(D) Quantification of wing shape (i) and area (ii). Anisotropy of the wing, measured in natural strain variables L - H, increases from 4 hours to 5 hours APF and remains roughly constant after 5 hours. Wing area increases slightly from 4 to 6 hours APF and increases greatly from 6 to 7 hours APF.

(E) Quantification of shape (i) and area (ii) of wing epithelia segmented cells. (A i) Average cell elongation $< Q_{xx} >$ increases sharply from 4 to 5 hours APF, indicating the cells are elongated along the anterior-posterior axis at the wing disc at 4 hours become almost isotropic at 5 hours APF. Cell areas are approximately constant from 4 to 5 hours APF, increase slightly from 5 to 6 hours APF, and sharply from 6 to 7 hours APF.

(F) The increase in cell area is sufficient to explain the increase in wing area. Normalised cell areas, (A_c /A_c^0), increase linearly with normalised wing areas, (A_w /A_w). A_c, cell area; A_c^0, average cell area at 5 hours APF; A_w, wing areas; A_w^0, average wing area at 5 hours APF. Each point (colour coded according to development time) corresponds to a single wing disc sample.

(G) The contribution of topological transitions to the overall convergent extension of the wing given by $R_{xx} = \frac{1}{2}\frac{d(L-H)}{dt} - \frac{d\langle Q_{xx}\rangle}{dt}$. R_{xx} is most pronounced from 4 to 5 hours APF and accounts for approximately half of the increase in anisotropy of the wing, the other half being due to change in cell elongation (see figure S3 and Supplemental Material and Methods). The data was obtained by averaging 2, 3, 6 and 2 fixed wings disc samples at 4, 5, 6 and 7 hours APF, respectively. The error bars correspond to standard deviation among wing discs at the same time point in development.

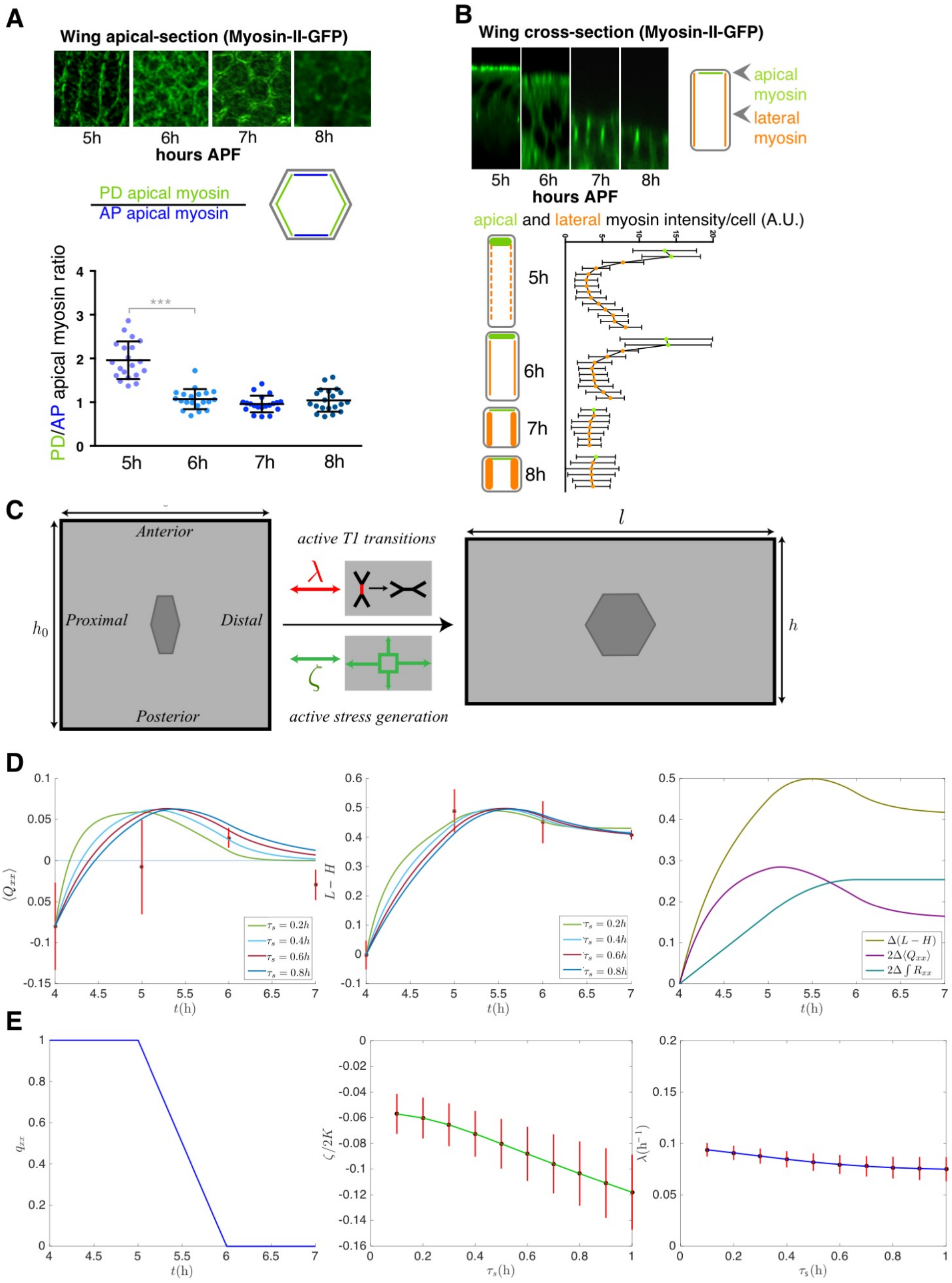

Figure S3. Quantification of Myosin-II localisation and a continuum model of convergent extension in the pupal wing (see Supplemental Material and Methods). Related to Figure 3 and 4.

(A) Measurements of Myosin-II-GFP fluorescence intensity were taken in Fiji at various points around the apical cell-cell junction of wing epithelia at the times shown. The ratio of myosin levels along the proximal-distal (PD) axis and anterior-posterior (AP) axis of the wing was calculated and plotted for multiple samples and time points. Note the 2-fold initial polarisation of Myosin-II-GFP along the proximal-distal axis at 5 hours (h) APF, which later dissipates. n=20 for each developmental stage. Average, standard deviation, and individual data points are represented. Statistically significant differences are indicated (***p<0.001).

(B) Measurements of Myosin-II-GFP fluorescence intensity were taken in Fiji at various points along the apical-basal axis of wing epithelia at the times shown. Data were plotted as intensity levels coloured by apical versus basolateral localisation as shown. At the end of convergent extension (5 to 6 hours APF), the apical surface shows the maximum values for Myosin-II-GFP fluorescence, whereas at the end of expansion and flattening, fluorescence levels are equally distributed along the apico-basal axis. n=20 for each developmental stage. Average and standard deviation are represented.

(C) The wing is approximated by a rectangular piece of tissue that deforms over time. Active stress generation due to polarised myosin in the tissue and oriented active topological transitions are responsible for the anisotropic growth of the wing.

(D) Time evolution of (*left*) the average cell anisotropy ($\langle Q_{xx}\rangle$) and (*centre*) the wing anisotropy $(L-H)$ obtained from a model of tissue deformation. Theory curves are fitted to experimental data (black points with red standard deviation bars) by taking different values for τ_s, the viscoelastic relaxation time of the tissue. *(right)* The contributions of cell shape change ($2\Delta\langle Q_{xx}\rangle$) and topological transitions ($2\Delta\int R_{xx}$) to the overall change in wing anisotropy $\Delta(L-H)$ for $\tau_s = 0.4$ h.

(E) (*left*) Time evolution of cell polarity q_{xx} taken for the fit, (*centre*) fitted parameter values of $\frac{\zeta}{2K}$ and (*right*) λ for a range of τ_s (red bars, standard error).

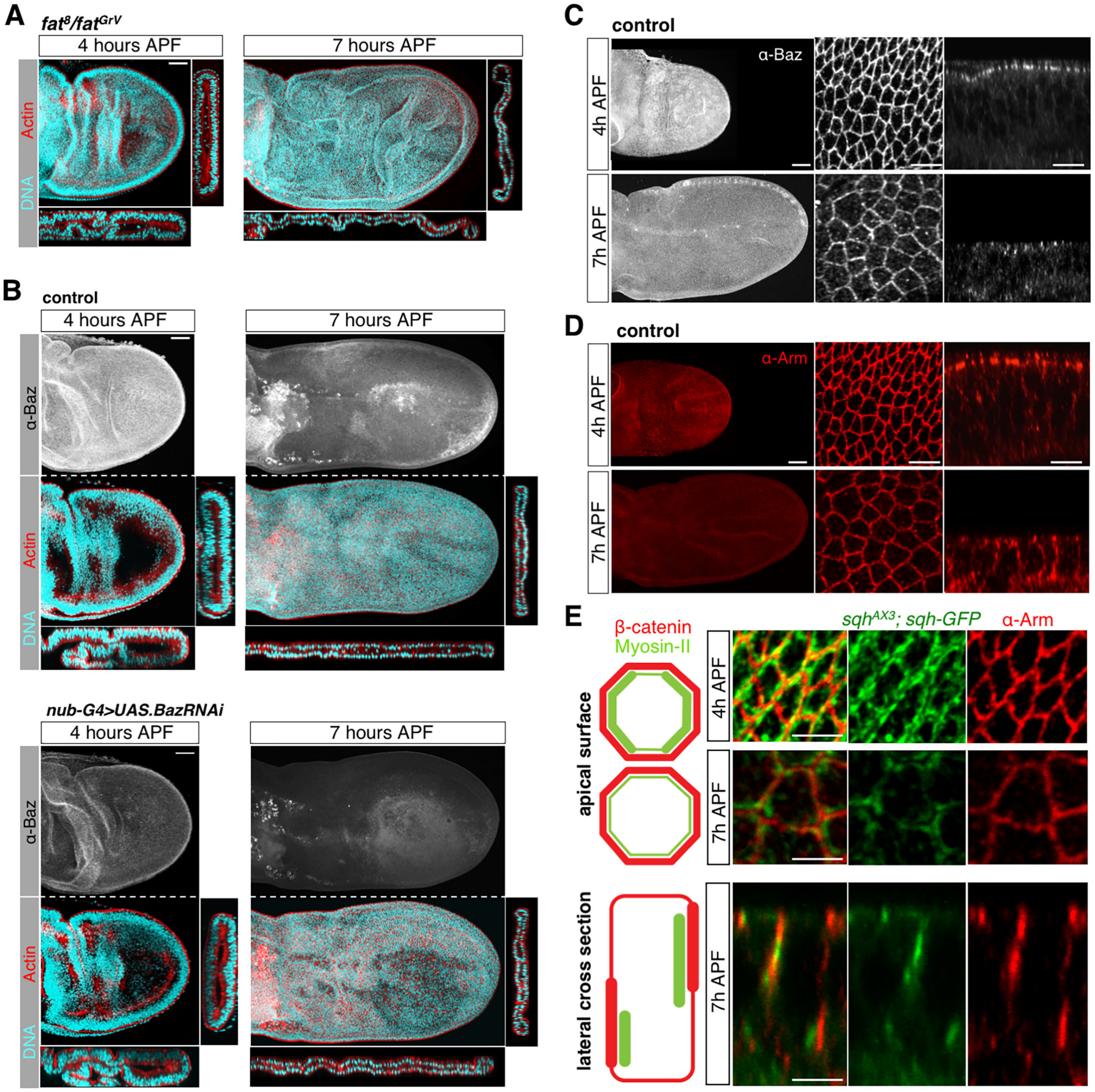

Figure S4. The Fat cadherin system and the polarity protein Bazooka are not essential for anisotropic extension of the pupal wing. Related to Figure 4.

(A) Inactivation of the Ds/Fat/Dachs planar polarity system in the *fat*^{8}/*fat*^{GrV} mutant (top). The *fat* mutant wings are already rounded prior to expansion and elongation, indicating that the key role of this planar polarity pathway is during larval stages of development, rather than during pupal stages. Scale bar 50 μm.

(B) Depletion of Bazooka/Par6 (Baz) in *nub-G4>UAS.bazRNAi* decreases Bazooka protein levels in the wing but does not affect anisotropic elongation of the epithelia. Control (top) and *nub-G4>UAS.bazRNAi* (bottom) wings showing Baz immunostaining in grey, actin in red and nuclei (DNA) in blue. Scale bars are 50 μm.

(C) In contrast to the elongating embryo, in which Bazooka/Par6 is planar polarised (Zallen and Wieschaus, 2004), Baz is not planar polarised during wing elongation, where it instead localises to adherens junctions. Scale bars are 50 μm (left) and 10 μm (middle and right).

(D) Armadillo/beta-catenin (Arm) co-localises with Bazooka in an apical ring and also localises laterally during expansion at 7 hours APF. Scale bars are 50 μm (left) and 10 μm (middle and right). Scale bars are 50 μm.

(E) Myosin-II localises adjacent to the adherens junctions marked by Arm both at the apical surface at 4 hours APF, and at the lateral membrane from 7 hours APF. Scale bar, 10 μm.

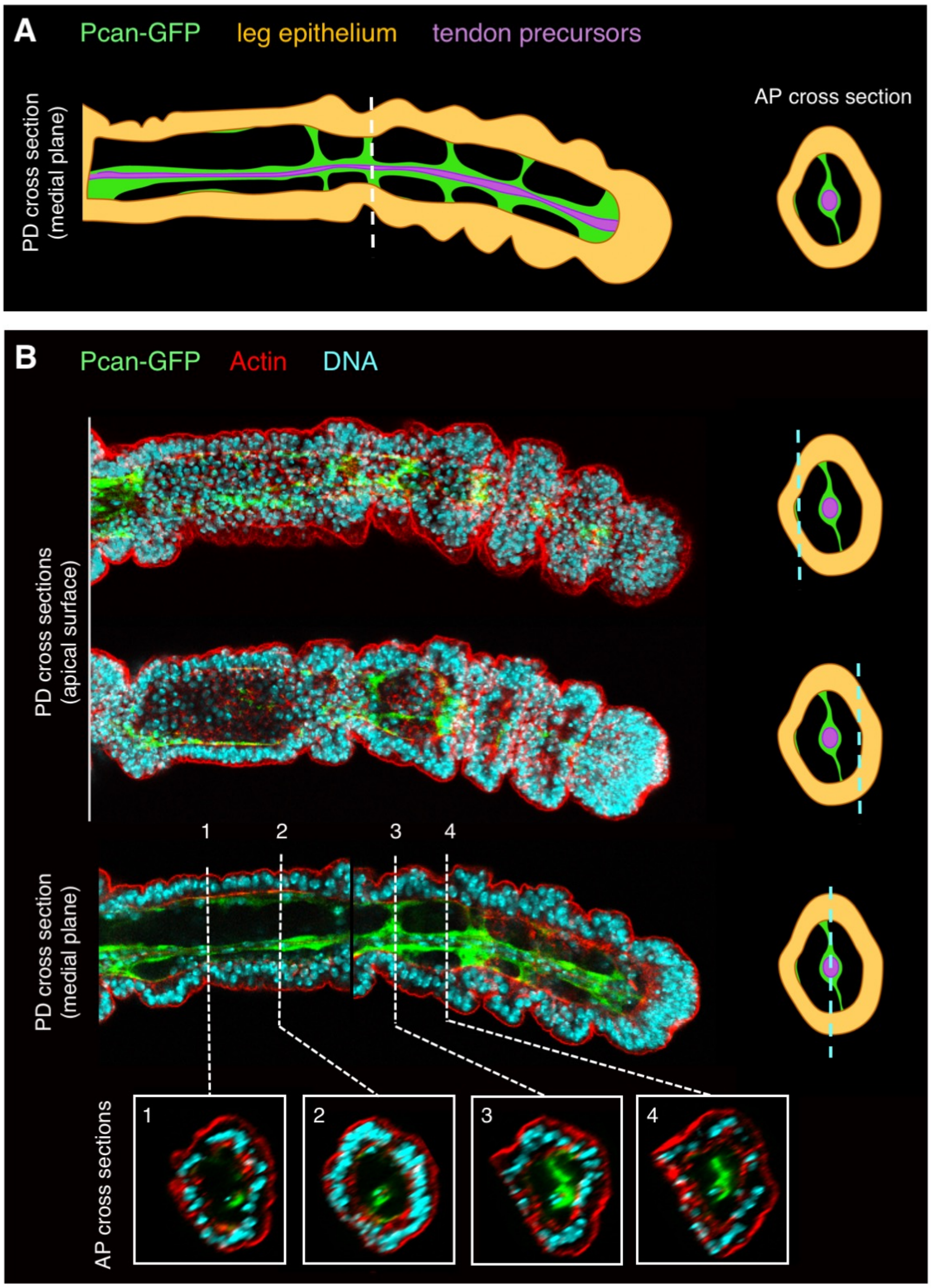

Figure S5. Perlecan localisation in 7 hours APF legs. Related to Figure 6.
(A) Diagram of a 7 hours APF leg. A dense layer of Perlecan (Pcan-GFP, green) covers the long tendon precursor that runs within the appendage (purple), but is less abundant at the basal side of the leg epithelium (yellow).
(B) Proximal-distal (PD) and anterior-posterior (AP) cross sections of 7 hours APF developing leg expressing *Pcan-GFP*. Traces of Pcan-GFP are present in the apical surface of the leg epithelium, but mostly accumulates surrounding then tendon precursors.

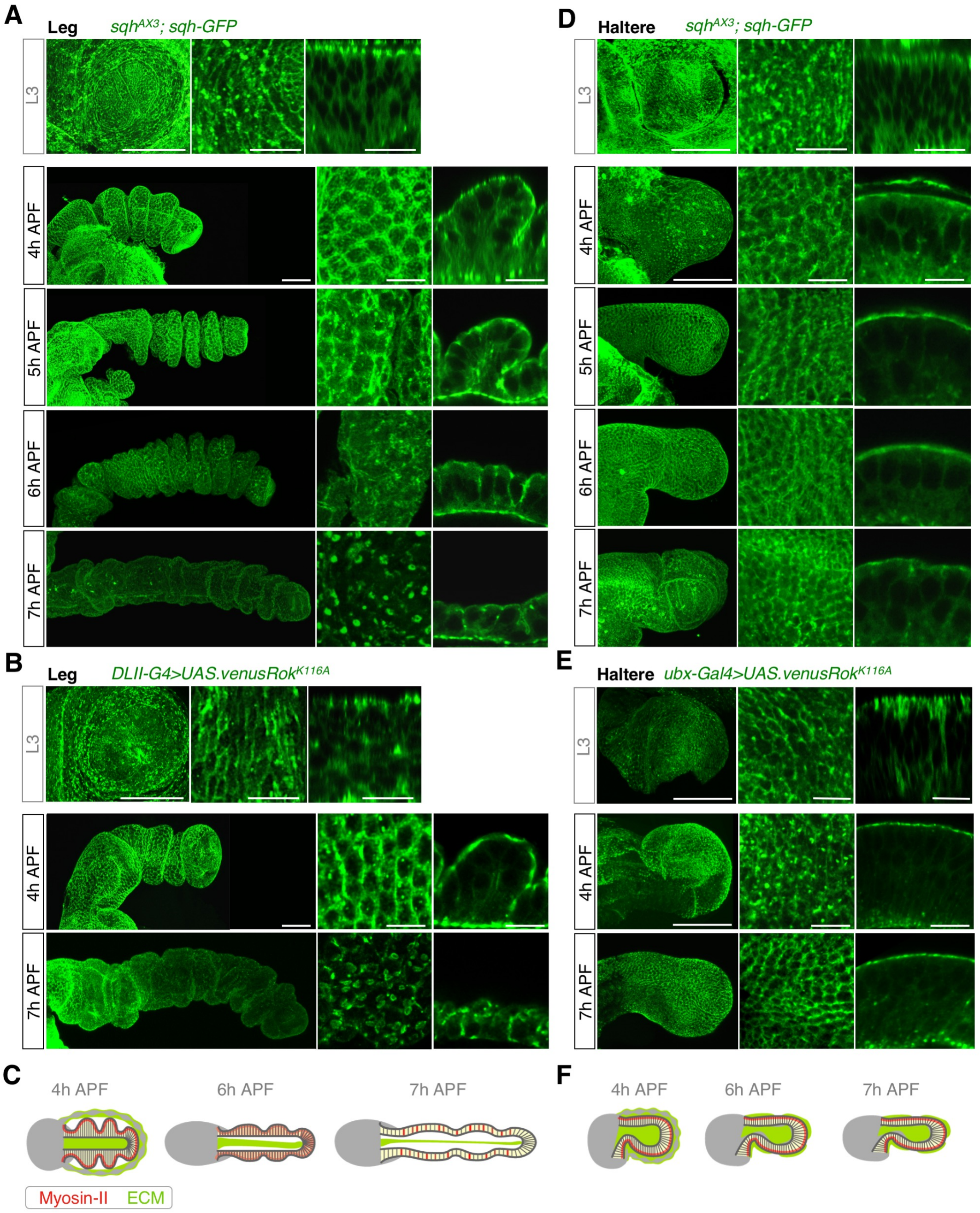

Figure S6. Myosin-II localisation in the leg and the haltere. Related to Figures 4, 5 and 6.

(A) Myosin-II-GFP localisation in the developing *Drosophila* leg. Maximum projections (left), high-magnification apical view XY-sections (middle) and lateral view Z-sections (right) of *sqhAX3; {sqh-GFP}* imaginal discs are shown. As in the wing, apical Myosin-II re-localises laterally in the leg epithelia from 5 to 7 hours APF.

(B) Similarly to Myosin-II, GFP-Rok is planar polarised in the leg at late larval stages, but localises laterally during the expansion phase (from 6 to 8 hours AF). Scale bars: 50µm (left) and 10µm (mid and right).

(C) Schematic diagram of leg disc morphogenesis from at 4 to 7 hours APF in sagittal cross sections. Leg epithelia is shown in yellow and peripodial membrane in grey. Apical and basal ECM is shown in green, and Myosin-II in red. Similarly to the wing, after peripodial membrane release and apical and basal ECM degradation, Myosin-II starts to re-localise from apical to the lateral plasma membrane, that, together with the unfolding of the leg, contributes to the flattening of the epithelia and the elongation of the leg discs.

(D) Myosin-II-GFP localisation in the developing *Drosophila* haltere. Maximum projections (left), high-magnification apical view XY-sections (middle) and lateral view Z-sections (right) of *sqhAX3; {sqh-GFP}* imaginal discs are shown. In contrast with wing and leg imaginal discs, Myosin-II remains apical in the haltere from L3 to 7 hours APF.

(E) Similarly to Myosin-II, GFP-Rok remains apical in the haltere from L3 to 7 hours APF.

(F) Schematic diagram of haltere disc morphogenesis from at 4 hours to 7 hours APF in sagittal cross sections. Haltere epithelia is shown in yellow and peripodial membrane in grey. Apical and basal ECM is shown in green, and Myosin II in red. In contrast with the wing and the leg, Myosin-II is localised apically from 4 to 7 hours APF, the epithelia barely flatten and the entire structure does not elongate.

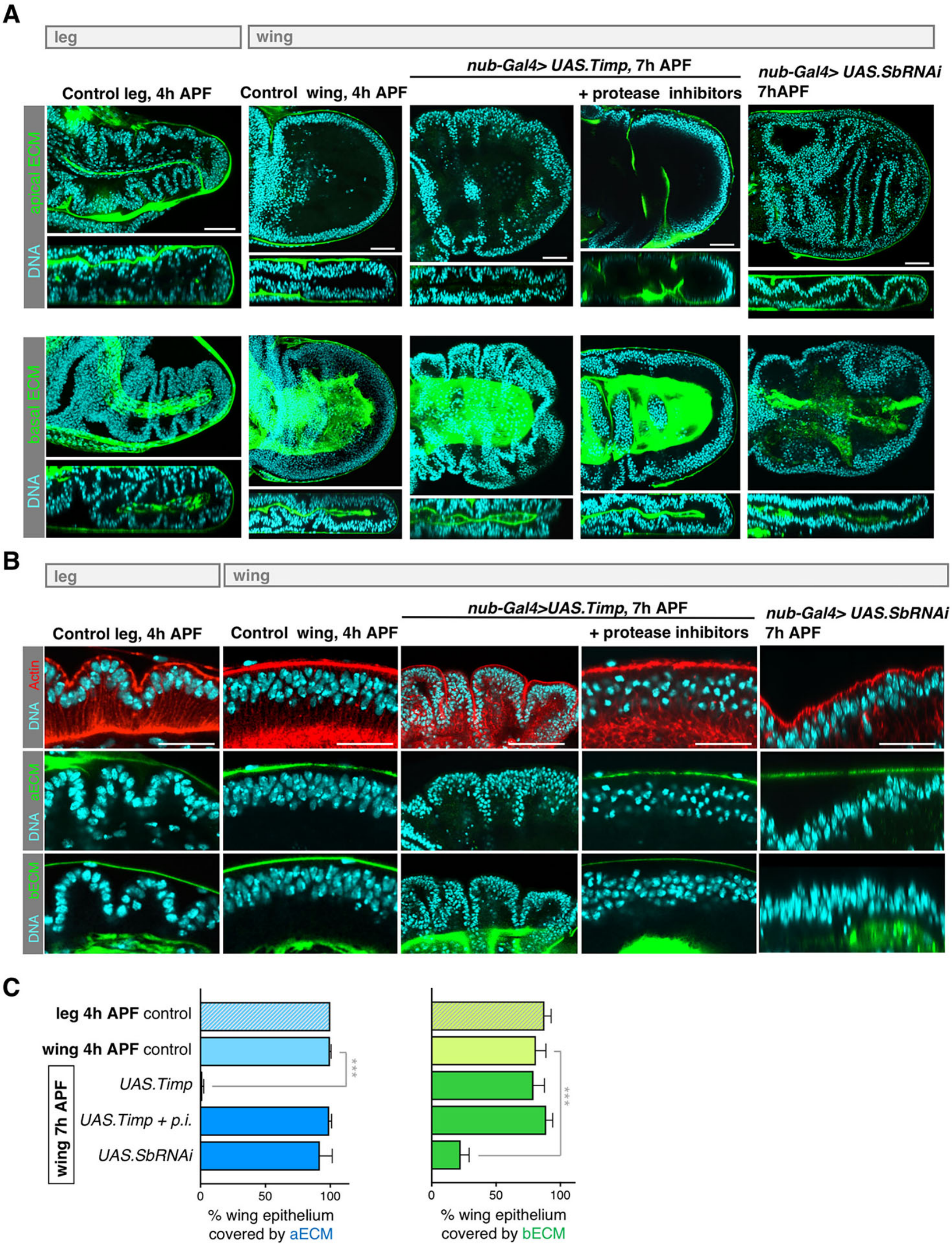

Figure S7. Cellular attachment to the basal ECM and concomitant detachment from the apical ECM leads to the folding of the epithelia. Related to Figure 1, 2 and 5.

(A) Cross sections of 4 hours APF control leg and wing, 7 hours APF *Timp* overexpressing wings (*dp-YFP nub-Gal4>UAS.Timp*) with or without protease inhibitor treatment, and *Stubble* overexpressing wing (*nub-Gal4>UAS.SbRNAi*). *Timp* overexpression does not affect basal Dumpy-YFP release but completely inhibits basal Collagen IV-GFP degradation, which impairs wing expansion and folds the epithelia. Protease inhibitors impair Dumpy release in *Timp* overexpressing wings and rescues the epithelial folding. Scale bar, 50 µm.

(B) High magnification of folded epithelia in 4 hours APF control leg and 7 hours APF wings overexpressing *Timp* or *Stubble*, and straight columnar epithelia in 4 hours APF control wing and 7 hours APF *Timp* overexpressing wings treated with protease inhibitors. When cells are attached to the apical but not the basal ECM it results in the folding of the epithelia. Scale bar, 25 µm.

(C) Quantification of the percentage of epithelia covered with apical Dumpy-YFP and basal Collagen IV-GFP in the different tissues and experimental conditions shown in (A,B). Average and standard deviation are represented, n≥4 for each developmental stage. Statistically significant differences between the control and the experimental conditions are indicated (***$p<0.001$).
